# Metal Halide Perovskites for High‐Energy Radiation Detection

**DOI:** 10.1002/advs.202002098

**Published:** 2020-10-11

**Authors:** George Kakavelakis, Murali Gedda, Apostolis Panagiotopoulos, Emmanuel Kymakis, Thomas D. Anthopoulos, Konstantinos Petridis

**Affiliations:** ^1^ Cambridge Graphene Centre University of Cambridge 9 JJ Thomson Avenue Cambridge CB3 0FA UK; ^2^ King Abdullah University of Science and Technology (KAUST) KAUST Solar Center (KSC) Thuwal 23955‐6900 Saudi Arabia; ^3^ Department of Electrical and Computer Engineering Hellenic Mediterranean University Estavromenos, PO Box 1939 Heraklion Crete GR‐71 004 Greece; ^4^ Department of Electrical and Computer Engineering Hellenic Mediterranean University Heraklion Crete GR 71500 Greece; ^5^ Institute of Emerging Technologies (I‐EMERGE) Hellenic Mediterranean University Research Center (HMURC) Heraklion Crete 71410 Greece; ^6^ Department of Electronic Engineering Hellenic Mediterranean University Chalepa Chania Crete 731 33 Greece

**Keywords:** high‐energy detectors, metal halide perovskites, perovskite X‐ray detectors, X‐ray imagers, *γ*‐ray detectors

## Abstract

Metal halide perovskites (MHPs) have emerged as a frontrunner semiconductor technology for application in third generation photovoltaics while simultaneously making significant strides in other areas of optoelectronics. Photodetectors are one of the latest additions in an expanding list of applications of this fascinating family of materials. The extensive range of possible inorganic and hybrid perovskites coupled with their processing versatility and ability to convert external stimuli into easily measurable optical/electrical signals makes them an auspicious sensing element even for the high‐energy domain of the electromagnetic spectrum. Key to this is the ability of MHPs to accommodate heavy elements while being able to form large, high‐quality crystals and polycrystalline layers, making them one of the most promising emerging X‐ray and *γ*‐ray detector technologies. Here, the fundamental principles of high‐energy radiation detection are reviewed with emphasis on recent progress in the emerging and fascinating field of metal halide perovskite‐based X‐ray and *γ*‐ray detectors. The review starts with a discussion of the basic principles of high‐energy radiation detection with focus on key performance metrics followed by a comprehensive summary of the recent progress in the field of perovskite‐based detectors. The article concludes with a discussion of the remaining challenges and future perspectives.

## Graphical abstract

Metal halide perovskites have emerged as a promising family of electronic materials for application in optoelectronics. One area where scientific interest in these materials has been intensifying is high‐energy radiation detection. This review discusses the progress in the application of metal halide perovskites for direct & indirect X‐ray and Gamma‐ray detection.

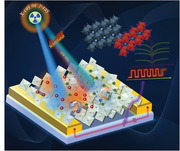

## 1 Introduction

**Figure 1:**
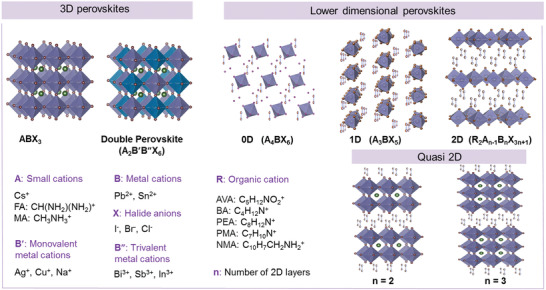
Schematic representation of the structures of 3D (simple and double), 0D, 1D, 2D, and quasi‐2D perovskites. Also shown are some examples of commonly used metal and organic cations.

Perovskites encompass a large family of materials, usually described by the general chemical formula ABX_3_.^[^ ^1^ ^]^ The first perovskite structure material was the calcium titanium oxide mineral (CaTiO_3_), discovered by Gustav Rose in 1839, and named after the Russian mineralogist Lev Perovski.^[^ ^2^ ^]^ Perovskites are divided into subgroups depending on their chemical composition and structure.^[^ ^3^, ^4^ ^]^ In traditional 3D ABX_3_ perovskites, the B‐site element is octahedrally coordinated in a BX_6_ configuration. The A component is situated within the cuboctahedral cavity formed by nearest‐neighbor X atoms in an AX_12_ polyhedron.^[^ ^5^ ^]^ Perovskites can also exist in a layered form, often termed 2D, with the ABX_3_ structure separated by thin sheets of spacer material. Synthetic versions of perovskite materials involving organic as well as inorganic cations have also been developed and exploited for a range of applications (**Figure** **1**).^[^ ^6^, ^7^, ^8^ ^]^

The structure of 3D perovskite can be described by the cubic contractual formula of A^+1^M^+2^(X^−1^)_3_, where each A (an organic group or an inorganic cation) has twelve neighboring X (halide atoms), and each M (a metal cation) connects with six adjacent X through ionic bonds. When a suitable organic molecule is employed as the A cation (e.g., MA^+^ (methylammonium): CH_3_NH_3_^+^ or FA^+^ (formamidinium):CH(NH_2_)_2_^+^), the resulting material is an inorganic–organic hybrid metal halide perovskite (MHP) (Figure 1). On the other hand, if the A cation is an inorganic atom, such as cesium (Cs^+^), the resulting compound is an inorganic MHP.

The tolerance factor (*t*), a parameter first introduced from Goldschmidt^[^ ^9^ ^]^ in 1926, is often used to predict the stability of the perovskite lattice on the basis of the ionic radii (*r*) of A (*r*_A_), B (*r*_B_), and X (*r*_X_), and is given as

(1) $$\begin{array}{c} t=\left(r_{A}+r_{X}\right)/\left[\sqrt{2}\left(r_{B}+r_{X}\right)\right] \end{array}$$

For a perfectly cubic perovskite lattice, *t* is close to 1. Empirically, the majority of MHPs synthesized to date form in the range 0.81 ≤ *t* ≤ 1.0. Hexagonal structures are typically formed when *t* > 1, and nonperovskite structures are formed when *t* ≤ 0.8.^[^ ^10^ ^]^ Besides *t*, the octahedral factor (*μ*), i.e., the ratio of the ionic radius of B site to the A site, provides a measure of the octahedral stability of the perovskite and is usually found in the range of 0.44 ≤ *μ* ≤ 0.9. The combination of those two factors defines the important parameter space for perovskite formability and stability.

The pioneering work by Miyasaka and co‐workers in 2009 laid the foundation for perovskite‐based photovoltaic (PV) research and kick‐started a scientific revolution on solar energy materials for use in third generation PV technologies.^[^ ^11^ ^]^ Since then, synthetic perovskites, and in particular MHP materials, have attracted enormous attention primarily due to their excellent optoelectronic properties including simple and flexible chemistry, bipolar charge conductivity, long diffusion carrier lengths (up to 175 µm), direct and tunable bandgap, high absorption coefficients, high quantum yield, and unmatched processing versatility. The very same properties are now propelling MHPs at the forefront of research and development in other scientific and engineering fields, one of which is optoelectronics.^[^ ^12^ ^]^

In the early days of perovskite research, the MHPs were utilized as a thin light absorber in a dye‐sensitized solar cell with power conversion efficiency (PCE) of ≈3.8%.^[^ ^11^ ^]^ However, the cells exhibited poor stability due to the liquid electrolyte‐based device structure. A few years later, researchers from different laboratories around the world replaced the liquid electrolyte with solid‐state hole conductors to overcome the stability issue and produced all‐solid‐state solar cells.^[^ ^13^, ^14^ ^]^ Since then, the research effort has been increasingly focusing on improving not only the PCE but also the stability of MHP‐based PVs. This effort has recently culminated in the demonstration of MHP PVs with a certified PCE in excess of 25.2% for single‐junction cells and over 29.15% for silicon/MHP tandem cells.^[^ ^15^ ^]^ Increasing the efficiency from 3.8% to 22.2% in approximately a decade is a remarkable achievement if one considers that analogous progress in traditional technologies, such as Si and GaAs solar cells, took 20 to 40 years to materialize. The tremendous progress is reflected in the fact that to date, the reported PCEs for polycrystalline MHPs PVs are inferior only to devices based on single‐crystal semiconductors such as Si and GaAs.^[^ ^15^ ^]^ The main concern regarding MHPs for PV applications right now is their apparently inherent chemical and structural instability under ambient conditions, a topic that is currently attracting increasing attention.^[^ ^16^, ^17^ ^]^

Looking beyond PVs, MHPs continue to demonstrate the huge potential for application in a wider range of opto/electronics including lasers,^[^ ^18^ ^]^ light‐emitting diodes,^[^ ^19^ ^]^ photodetectors,^[^ ^20^ ^]^ sensors,^[^ ^21^, ^22^, ^23^ ^]^ and particle detectors,^[^ ^24^, ^25^, ^26^, ^27^ ^]^ to name a few. In the area of sensing applications, MHPs have demonstrated the ability to transduce different environmental stimuli into an optical or electrical signal, which can be seen as an added advantage for applications in the field of sensing.^[^ ^28^, ^29^, ^30^, ^31^, ^32^ ^]^ For instance, various physical properties of MHPs, such as photoluminescence, optical spectra response, and electrical conductivity, can be modulated upon exposure to external stimuli such as gasses^[^ ^29^, ^31^ ^]^ and light.^[^ ^33^ ^]^ Moreover, MHPs sensors can be self‐powered and able to operate at room temperature, exhibit high sensitivity with fast response and recovery times, while being manufactured via simple and scalable techniques.

**Figure 2:**
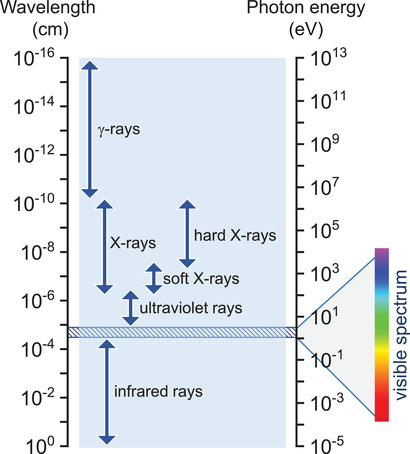
The electromagnetic spectrum between infrared (IR) and gamma rays, and their respective wavelengths and photon energies. The range of the visible (vis) wavelengths is magnified for clarity.

The attractive properties of MHPs have also been exploited for applications in X‐ray detectors.^[^ ^34^, ^35^ ^]^ Since the discovery of the X‐rays,^[^ ^36^ ^]^ there has been intense effort to develop efficient large‐area X‐ray detectors for applications ranging from crystallography^[^ ^37^ ^]^ and medicine,^[^ ^38^ ^]^ to space exploration.^[^ ^39^ ^]^ The same is true for *γ*‐rays (0.1–10 MeV) (**Figure** **2**), which are usually emitted by radioactive materials and their detection is essential for various security applications, including radiological security, nuclear defense,^[^ ^40^ ^]^ but also radioactive isotope identification. Thus, the development of high energy photon detector technologies that combine improved performance with lower‐cost and the ability for scalable, large‐area, high‐throughput manufacturing, could pave the way to new products with numerous benefits to our society.

Solid‐state X/*γ*‐ray detectors rely on effective interaction between the high‐energy photons and the electrons in the atoms of the materials used to construct the detector, which in turn leads to the generation of an electrical or optical signal. The magnitude of the interaction depends largely on the elemental composition of the detector material, its crystallinity, its density/absorption coefficient, the photon intensity (dose rate), direction with respect to the orientation of the detector material, phase, and energy.^[^ ^41^, ^42^ ^]^ The detection process should be as efficient as possible since it reduces the need for harmful high radiation doses, which is particularly relevant for medical imaging applications where the health and safety of patients are of paramount importance.

To date, all commercial X‐ray detection systems, based either on direct detection (e.g., silicon (Si),^[^ ^43^ ^]^ amorphous selenium (*α*‐Se),^[^ ^44^ ^]^ lead iodide (PbI_2_),^[^ ^45^ ^]^ mercury(II) iodide (HgI_2_),^[^ ^46^ ^]^ cadmium zinc telluride (Cd_1−_ *_x_*Zn*_x_*Te),^[^ ^47^ ^]^ thallium(I) bromide (TlBr)) or indirect scintillator‐based systems (thallium‐activated cesium iodide CsI(Tl),^[^ ^48^ ^]^ thallium‐activated sodium iodide NaI(Tl), etc.), suffer from low sensitivity, low X‐ray absorption cross‐section (e.g., *α*‐Se for photon energies >50 keV), various material instabilities (e.g., PbI_2_ and HgI_2_), high charge trap density (e.g., Cd_1−_ *_x_*Zn*_x_*Te), limited spatial resolution (e.g., scintillator systems based on CsI(Tl)), high processing temperature required during the material crystallization processes (e.g., Si, Cd(Zn)Te), and difficulty in tuning their radioluminescence across the visible spectrum (Figure 2). As already mentioned, in the field of medical imaging such as computed tomography (CT), the use of inefficient detectors leads to the need for higher radiation doses, which in turn increases the risk for patients due to longer radiation exposure requirements. These are the reasons why the development of new X‐ray and *γ*‐ray detector technologies that combine improved performance with other attractive attributes such as compactness and large‐area features, with unusual form factor, have been attracting increasing attention.

**Figure 3:**
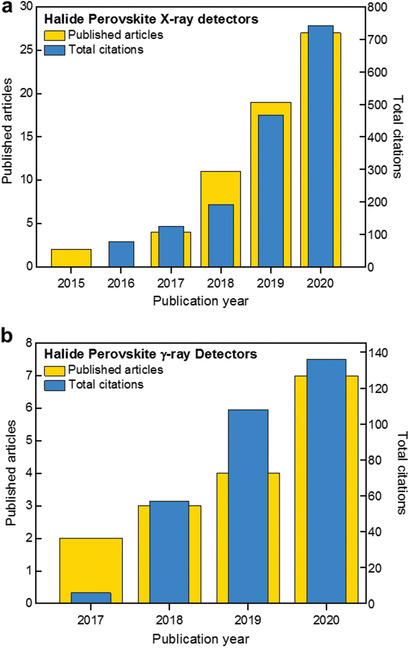
Illustration of the increasing number of published articles and total citations for the period between 2015 and 2020 for searches containing the keywords a) X‐ray detector and halide perovskite, and b) *γ*‐ray detector and halide perovskite either in the title or abstract. Source: Scopus bibliographic database (July 2020).

Since the pioneering work by Stoumpos et al. in 2013^[^ ^49^ ^]^ on direct solid‐state X‐ray detectors, the field of high‐energy radiation detectors based on MHP has flourished, with the number of published studies increasing exponentially over the years (**Figure** **3**). It is now evident that MHPs have the potential to address most of the aforementioned scientific and technological shortcomings that incumbent technologies face.^[^ ^50^ ^]^

Besides, MHPs offer processing versatility that is absent from commercial technologies and which in the future could lead to better performing and more affordable commercial products. For instance, MHPs can be processed using upscalable techniques, such as ink‐jet printing or slot‐die coating, at low temperatures and over large area substrates, which can then be used to fabricate highly efficient and sensitive detectors with unusual physical characteristics.^[^ ^51^ ^]^ In this article, we aim to provide the reader first with an introduction to the basic principles of high‐energy radiation detection, and second, with a critical review of the progress achieved to date in the rapidly advancing area of X‐ray and *γ*‐ray detectors based on MHPs. Technologies covered include direct and indirect high‐energy photon detectors, with emphasis on the remaining technological hurdles and open questions. The review concludes with a summary and perspective of future developments.

## 2 High‐Energy Radiation Detectors

There are two primary types of high‐energy radiation detectors and are classified depending on the detection principle: I) direct detectors, and II) indirect detectors.^[^ ^52^ ^]^ Direct detectors rely on photoconductive materials that are sensitive to particular high energy radiation. On the other hand, indirect detectors employ scintillator materials that convert, in energy, the absorbed high energy X‐rays (0.1–100 keV) or *γ*‐rays (0.1–10 MeV) (Figure 2) to ultraviolet light (UV) or visible (vis), which is subsequently detected by standard photodiode/array.^[^ ^53^, ^54^, ^55^ ^]^ Depending on the scintillator material used, the emitted light may be in the ultraviolet or visible part of the electromagnetic spectrum. On the other hand, direct detection of X‐ray or *γ*‐rays relies on the collection of charges generated within the device's active layer upon photon absorption. The generated electrical signal is recorded using external circuitry, which can subsequently be analyzed to generate a digital image. Next, we discuss the pros and cons of each detection approach.

### 2.1 X‐Ray Detectors

#### 2.1.1 Direct X‐Ray Detection

The operating principle of direct high‐energy radiation detectors is based on the direct interaction of the incoming X‐ray photons with the sensing material, typically a semiconductor, and the instant generation of an electrical signal (current or voltage). The interaction between the soft X‐ray photons (Figure 2) and the sensing material relies on photoelectric absorption. In contrast, the detection of hard X‐ray photons is due to Compton scattering (photon–electron interaction). Absorption of high‐energy photons results in the generation of electron–hole pairs that are subsequently collected by the specially designed device electrodes due to the application of an external electric field.^[^ ^50^, ^56^ ^]^ Important figures of merit that determine the performance of a direct conversion X‐ray detector include:

1) Mass attenuation coefficient of the photoactive material. It characterizes how easily the sensing material can be penetrated by a beam of high‐energy photons.
2) Density of the sensing material. It depends on the elemental composition and structural properties/morphology of the active material.
3) Spatial resolution of the detector. It determines the image resolution and hence its sharpness. The higher the spatial resolution (i.e., smaller pixel size), the higher the image quality.
4) Signal‐to‐noise ratio (SNR). It defines the strength of the electrical/optical signal upon excitation to that of unwanted signal (noise such as dark current).
5) Response time. It is defined as the time taken for the detector to respond to an external stimuli, i.e., pulse of X‐ray photons. Fast response helps to minimize the exposure duration time and/or enable higher frame rate during imaging and is important for medical imaging applications.
6) Structural uniformity of the sensing material layer. It is determined, primarily, by the processing versatility of the active material employed.
7) Operational stability. It relates to the detector's ability to maintain a specific level of performance during operation.
8) The electron/hole mobility (*μ*_h/e_)–lifetime (*τ*) product (*μ*_h/e_ × *τ*). It is a key parameter and highlights the quality of a semiconductor X‐ray detector. The larger the *µτ* product is, the higher the charge collection efficiency of the detector and its performance.

Broadly speaking, the larger the interaction between the incoming high energy photon and the semiconductor material (also known as the stopping power), the better the performance of the detector. The stopping power, which is defined as the rate of energy lost per unit of path length (*x*) by a charged particle with kinetic energy (*T*_E_) in a medium of atomic number *Z* (*Z* ∝ *ρ*, where *ρ* is the density of the material), is represented as

(2) $$\begin{array}{c} dT_{E}/\rho dx\;\mathrm{MeV}\;cm^{-1}\;\mathrm{or}\;J\;m^{-1} \end{array}$$

Another critical parameter is the ionization energy (*W*) of the sensing material and is defined as the total adsorbed energy divided by the total number of extracted electrons. The minimum ionization energy is a function of the energy bandgap (*E*_g_) of the absorbing material employed and according to Que and Rowlands,^[^ ^57^ ^]^ it can be estimated using

(3) $$\begin{array}{c} W=2.2E_{g}+E_{\mathrm{photon}} \end{array}$$

or

(4) $$\begin{array}{c} W=3E_{g} \end{array}$$

High detection sensitivity is needed to generate high‐quality images with good contrast at a given X‐ray dose. Detectors with high sensitivity generate larger electrical signals that result in a higher SNR. The sensitivity of X‐ray detectors can be improved using two different approaches: a) increasing the *μ*_h/e_ × *τ* product within the sensing material as it relates to the carrier drift length (*L*_D_) given as *L*_D_ = *(k*_B_ *Tµτ*/*e*)^1/2^, which in turn governs charge carrier extraction; and b) increasing the applied reverse bias but often with adverse effects on the noise/dark current. The use of materials that combine high *μ*_h/e_ × *τ* product and exhibit low trap density is key for efficient direct X‐ray detectors.

The *μ*_h/e_ × *τ* product for a given sensing material can be derived by fitting the photoconductivity (*I*) curve using the modified Hecht formula^[^ ^58^ ^]^

(5) $$\begin{array}{c} I=\frac{I_{0}\mu \tau V}{L^{2}}\frac{1-\exp\left(-\frac{L^{2}}{\mu \tau V}\right)}{1+\frac{L}{V}\frac{s}{\mu }} \end{array}$$

where *I*_0_ is the saturated photocurrent, *L* is the material layer thickness, *V* is the applied bias, and *s* the surface recombination velocity. The sensitivity of the device, on the other hand, is related to the linear dynamic range (LDR) of the detector that describes the range of X‐ray dose rate under which the sensitivity remains constant. High LDR allows for more accurate measurements of the high energy radiation dose rate under a broad variation range. The sensitivity of the detector is calculated by

(6) $$\begin{array}{c} S=\frac{\int \left[I_{X-\mathrm{ray}}\left(t\right)-I_{\mathrm{dark}}\right]dt}{D\times V} \end{array}$$

where *I*_X‐ray_ and *I*_dark_ are the currents under X‐ray irradiation and in the dark, respectively, *D* is the dose, and *V* is the detector volume.

An additional requirement is that the thickness of the active layer should be at least three times larger than the attenuation length—defined as the distance over which at least 63% of high‐energy photons have been absorbed. Semiconductor‐based direct high energy X‐ray detectors operate in the current mode, where the magnitude of the generated electrical current is proportional to the incident photon energy. Direct high‐energy radiation detectors offer improved spatial resolution, one of the main advantages over indirect scintillating systems. Some of the key attractive features associated with each X‐ray detectors technology are listed in **Table** **1**.

**Table 1:** Advantageous characteristics associated with direct and indirect X‐ray detector technologies

Direct X‐ray detector	Indirect X‐ray detector
Simpler to manufacture No operational device thresholds Higher spatial resolution Simpler device operation	High operational stability No external bias required Lower manufacturing cost High temporal resolution

#### 2.1.2 Indirect X‐Ray Detection

Indirect X‐ray detection is the second approach often exploited to detect high energy X‐rays. Here, the incoming X‐ray photons are converted to UV or visible light by a scintillator. The generated radioluminescence is then detected by a photodiode, the electrical signal of which is recorded by an external circuit. The ability of scintillators to stop high‐energy photons and convert them to lower energy visible photons has found numerous applications in security, X‐ray astronomy, and medical imaging, to name but a few applications.^[^ ^57^, ^59^ ^]^

The interaction between high‐energy photons and the scintillator material can occur via three main processes; i) photoelectric absorption, ii) Compton scattering, and iii) electron and positron pair formation—also known as pair production for energies >1.022 MeV.^[^ ^42^, ^52^ ^]^ All processes are characterized by different absorption coefficients that are ultimately determined by the scintillator material's atomic number (*Z*), and the photon energy. In the photoelectric effect, the photon energy is fully absorbed by a bound electron, typically a core electron in the K‐ or L‐shell, which is then ejected into the vacuum, ionizing the host atom.^[^ ^60^ ^]^ When photoelectric effect dominates and for photon energies far from the absorption edge, the linear absorption coefficient (*μ_L_*) is given by

(7) $$\begin{array}{c} \mu_{L}\approx \rho Z^{n}/E^{3.5} \end{array}$$

where  *ρ* is the materials density, *E* is the photon's energy, and *n* is a constant, which typically varies between 3 and 4.

Compton scattering occurs at higher energies and is attributed to inelastic interactions between a (weakly) bound electron and the high energy photon. Here, part of the photon's energy is transferred to the electron with the exact amount depending on the scattering angle. The energy lost by the photon is gained by the scattering electron, which is excited to a higher energy state. The linear absorption coefficient (*μ*_C_) for Compton scattering is given by^[^ ^42^, ^43^ ^]^

(8) $$\begin{array}{c} \mu_{C}\approx \rho /{\left(E\right)}^{1/2} \end{array}$$

The process leads to the generation of lower energy exciton (loosely bound hole–electron pair) that is transported to defect states, or activators, within the scintillating material that ultimately recombines and generate visible photons. The emitted light can then be detected using different types of photodetectors such as Si photodiodes, thin‐film phototransistors (photo‐TFTs), photomultiplier tubes (PMTs), complementary metal–oxide–semiconductor (CMOS) photodetectors, silicon avalanche photodiodes, or charge‐coupled devices (CCDs) coupled with the scintillator element. One of the main strengths of indirect scintillator detectors is that they are of low production cost and more stable than direct high‐energy radiation detectors.^[^ ^42^, ^43^ ^]^

The most important figures of merit for scintillators include the light yield (LY), light decay time, and environmental stability. The LY describes the number of electron–hole pairs that are generated during the ionization process per unit energy

(9) $$\begin{array}{c} \mathrm{LY}=10^{6}SQ/\left(\beta E_{g}\right)\;\left(\mathrm{in}\;\mathrm{photons}\;\;\mathrm{Me}V^{-1}\right) \end{array}$$

where *S* is the efficiency of transport of electron–hole to the optical (emissive) center, *Q* is the luminescence efficiency, and *β* is a constant with a typical value of 2.5. The recombination centers in the scintillator could induce radiative but also nonradiative recombination (nonemissive and hence a lossy process) due to the presence of defects and/or impurities. The growth and use of single crystals as the scintillating medium can suppress the concentration of these defects (trap states) and improve the luminescence yield of the system while helping to accelerate the photoluminescent processes. Key figures of merit for scintillators include:

1) Radiation absorption efficiency. It relates to the absorption coefficient or absorption length and is mainly determined by the atomic number and density of the scintillator material used.
2) LY. It determines the efficiency, sensitivity, and energy resolution of the system.
3) Decay time. It is defined as the time taken from the absorption of high‐energy photons to the emission of optical photons. It is estimated by measuring the decay time of the scintillation optical emission signal upon excitation. Long response times result in undesired effects such as afterglow, which is characterized by a significantly longer lifetime.
4) The self‐absorption of light. This is an undesired effect and in order to minimize it, the thickness of the scintillator should be optimized in order to avoid reabsorption of the initially emitted optical photons.
5) Energy resolution. It describes the ability of the material to distinguish different radiation energies.
6) Emission wavelength. This is the emission spectrum of the visible light generated by the scintillator upon excitation. The latter should match the absorption spectrum of the photodiodes employed in the detector. A good match ensures minimal losses during the scintillation process.
7) Stability. It characterizes the chemical and radiation stability—also known as radiation hardness—of the scintillator material.
8) Proportionality. This describes the linearity of the detected signal in the detector to the incoming high energy radiation intensity.
9) Spatial resolution. This is also known as modulation transfer function (MTF) and determines the spatial frequency response of the photodetector.

### 2.2 Gamma‐Ray (*γ*) Detectors

Gamma‐ray (*γ*‐ray) detection is important for many applications, such as homeland security, medical imaging, nuclear inspection, astrophysical studies, and fundamental science.^[^ ^61^, ^62^ ^]^ There are three mechanisms that describe the interactions between *γ*‐ray photons with the detector material and depend on the photon energy. 1) The Photoelectric process, where the entire energy of the *γ*‐ray photon is transferred to electrons. The latter occurs when the photon's energy is between 10 and 500 keV. 2) The Compton scattering mechanism where part of the *γ*‐ray energy is lost and is transferred to the electrons. This process dominates for photons with energy in the range of 50 keV to 3 MeV. 3) The pair production in which an incoming *γ*‐ray photon with energy in excess of 1.022 MeV generates a positron and one electron.

Detection of *γ*‐rays is more demanding than X‐ray from a material point of view. The high energy of *γ*‐ray photons requires the use of semiconductors with the following important characteristics: a) composed of elements with high atomic number (*Z*) and hence large stopping power (see Equation (2)), b) exhibit large bipolar *μ*_h/e_ × *τ* product to enable efficient detection of the generated electrical signal, and c) is characterized by large bulk resistivity of >10^9^ Ω cm to enhance the SNR (smaller dark current). The photopeak energy resolution defined as the ratio between the full‐width‐at‐half‐maximum (FWHM) value of that photopeak at a specific energy.^[^ ^63^ ^]^ The energy resolution is very important as it allows the detector to distinguish between *γ*‐ray photons with a different energy.

Direct *γ*‐ray detection exploits photon counting or other spectroscopic techniques to be able to distinguish the presence of the specific radiative isotopes responsible for the photon emission.^[^ ^64^ ^]^ This type of detector operates in the voltage mode since the photon flux intensity is comparatively weak as the high‐energy photons arrive to the detector at different times.^[^ ^65^ ^]^ The detector system then performs an event by event analysis to sort out the intensity versus the energy of the detected gamma photons. This process yields a histogram of the energy‐resolved spectrum, which is a map of the electrical pulse height produced by *γ*‐photons interacting with the sensing material. Due to the low intensity of the signal, a charge‐sensitive preamplifier is often used to integrate the detector signal for a given interval of time. The collected charge is then converted into a voltage signal that is subsequently registered by the readout electronics.

In the case of indirect *γ*‐ray detection, incoming photons with energies higher than 1.02 MeV (twice the electron's rest‐mass energy) interact with the active material leading to pair generation. The latter is a relativistic phenomenon and the absorption coefficient (*μ*_P_) is expressed as

(10) $$\begin{array}{c} \mu_{P}\approx \rho Z\ln\left(2E/\left(m_{e}c^{2}\right)\right) \end{array}$$

where *m*_e_ is the mass of electrons, *c* is the speed of light and *ρ* is the material's density. In general, pair generation becomes the dominant light–matter interaction mechanism for photons with energies higher than 8 MeV.^[^ ^66^ ^]^ Photon absorption (i.e., the first stage of scintillation lasting ≈1 ps) is followed by the charge transport and energy transfer steps. At this stage of the scintillation process, the energy of hot electrons and holes is transferred to the luminescence centers, a step followed by visible light emission via the aforementioned process.

Commercially available direct *γ*‐ray semiconductor detectors, e.g., high purity germanium (HPGe),^[^ ^67^ ^]^ require cryogenic cooling to lower the dark current due to low bandgap of Ge (0.66 eV). On the other hand, semiconductors such as CZT (Cd_1−_ *_x_*Zn*_x_*Te for 0 < *x* < 0.2), suffer from low bulk resistivity of ≈10^9^ Ω cm,^[^ ^68^ ^]^ cost restricted crystal manufacturing, and incompatibility with readout circuits due to the high‐temperature crystal growth required. TlBr^[^ ^69^ ^]^ demonstrates the highest energy resolution at room temperature of 1%, as the material has a very high bulk resistivity of the order of 10^11^ Ω cm but suffers from a very low *μ*_e/h_ × *τ* product, while it is costly to produce. Importantly, *γ*‐rays can also be detected indirectly. Commercially available systems employ NaI(Tl) crystals^[^ ^70^ ^]^ and CsI(Tl) crystal scintillators.

From our discussion so far, it becomes evident that both direct and indirect X‐ray and *γ*‐ray detection schemes are characterized by certain limitations. For example, direct detectors rely primarily on polycrystalline materials that are known to suffer from long reaction times that enhance the ghosting effect due to the persistent electrical signal following high energy photon absorption. Use of single crystals could alleviate the problem, but the cost of manufacturing increases significantly. On the other hand, indirect detectors suffer from limited spatial resolution, which in turn lowers the accuracy of the detector. To this end, the scattering of the generated visible light within the relatively thick scintillating material is a major hurdle.

## 3 Metal Halide Perovskites for High‐Energy Radiation Detection

Research and development efforts toward next‐generation materials for the detection of high‐energy radiation have been intensified in recent years. The majority of the ongoing efforts are geared toward improving the manufacturability and sensitivity of the detection elements and systems.^[^ ^71^ ^]^ This is why the development and/or discovery of materials that combine key functionalities with inexpensive manufacturing has become a hot area of research with MHPs currently leading the way.^[^ ^10^, ^49^, ^50^, ^71^ ^]^ MHPs combine a high material density (≈4 g cm^−3^) due to their ability to incorporate atoms with large atomic number (*Z*), e.g., Cs (*Z* = 55), Pb (*Z* = 82), Sn (*Z* = 50), In (*Z* = 53), and Br (*Z* = 35). The need for compounds consisting of high *Z* atoms is dictated by the scaling of the X‐ray absorption strength given by *Z* ^4^/*AE* ^3^, where *A* is the atomic mass and *E* the energy of the high‐energy photons. High material density leads to large X‐ray absorption cross‐section and short penetration depths – typically on the order of hundreds of micrometers (µm). Additionally, MHPs offer large *μ*_e/h_ × *τ* products, short detection times (ns), and highly emissive triplet excited states with fast emission rates.

The processing versatility and the relatively high bulk resistivity of MHPs (typically ≈10^7^ Ω cm) represent a few additional important attributes with the prototypical methylammonium lead iodide (MAPbI_3_), having attracted most of the attention to date.^[^ ^72^, ^73^, ^74^, ^75^ ^]^ Furthermore, their lower cost, as compared to established material technologies, combined with MHPs’ simple chemistry, processability, the low charge trap density and their defect tolerant nature (i.e., *μ*_e/h_ × *τ* product), have propelled MHPs to become auspicious for applications related to high‐energy radiation detection.

**Figure 4:**
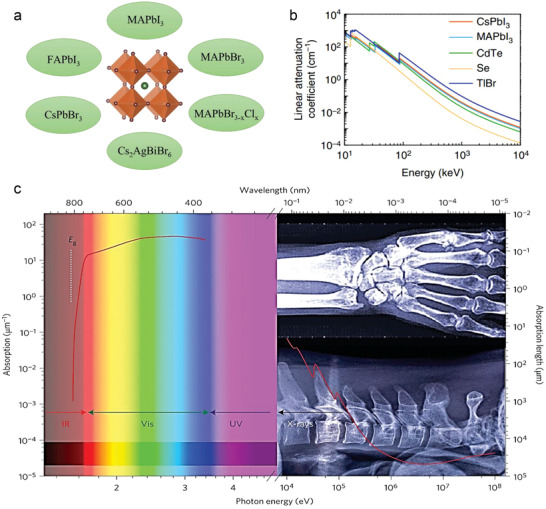
a) Metal halide perovskites commonly used in radiation detectors. b) The linear attenuation coefficient of CsPbI_3_, MAPbBr_3_, CdTe, Se, and TlBr versus photons energy. Reproduced with permission.^[^ ^50^ ^]^Copyright 2019, Springer Nature. c) Absorption coefficient and length versus photon energy from visible to hard X‐rays for MAPbI_3_. Reproduced with permission.^[^ ^78^ ^]^Copyright 2015, Springer Nature.

**Figure** **4**a,b shows the chemical composition of various MHPs and the attenuation coefficients for two commonly employed ones, namely, MAPbI_3_ and cesium lead iodide (CsPbI_3_), along those of commercial materials such as Se, TIBr, and CdTe, respectively.^[^ ^76^ ^]^ The stopping power for *γ*‐rays of halide perovskites (linear attenuation coefficient of 0.09 cm^−1^) is nearly two times higher than commercial deployed CdTe, further highlighting the potential advantages of the technology.^[^ ^77^ ^]^ Moreover, some MHPs (e.g., MAPbI_3_) offer extraordinary wide absorption that spans from the visible to hard X‐ray part of the electromagnetic spectrum making then an excellent choice for both direct and indirect high‐energy detector applications (Figure 4c). However, in the case of most studied MHPs, the reliance on toxic elements, such as Pb, represents a significant environmental challenge that would need to be addressed before commercial deployment. Strengths, weaknesses, opportunities, and threats (SWOT) analysis of both X‐ray and *γ*‐ray detectors are availed in **Table** **2**. Further discussion on this critical issue along recent developments in the field is provided later in this review. **Table** **3** summarizes the detailed performance of the current X‐ray detectors based on various perovskite materials.

**Table 2:** Strengths, weaknesses, opportunities, and threats (SWOT) analysis from a material point of view for metal halide perovskite‐based X‐ray and *γ*‐ray detector technologies

Strengths	Weaknesses
Scalable, large‐area processing Low temperature processing Simple device manufacturing High carrier mobility and long diffusion lengths (high *μ*_e/h_ × *τ*) Energy bandgap tunability Facile growth of high‐quality large crystals High X‐ray stopping power High X‐ray sensitivity	Poor environmental stability Material stability due to ion‐migration during operation Low bulk resistivity and high dark currents High cost due to high crystal quality required Long reaction times and ghosting effects
**Opportunities**	**Threats**
Compatible with flexible, large‐area X‐ and *γ*‐ray detectors Compatible with inexpensive, temperature‐sensitive substrate materials Realization of self‐powered, room temperature operated, high SNR and high sensitivity detectors Faster response times	Material toxicity Material and system recyclability Ability to process layers of high structural quality over large‐area substrates

**Table 3:** Summary of key performance indicators (KPI) of 3D MHP‐based X‐ray detectors reported to date

Perovskite	Key performance indicators	Publication year	Refs.
CH_3_NH_3_PbI_3_ single crystals	Charge collection efficiency of 75% for 20–35 keV X‐ray photons Attenuation coefficient of 14 cm^2^ g^−1^ High stability under X‐ray irradiation (<20% drop of the photocurrent for 40 Sv dose)	2015	^[^ ^81^ ^]^
CH_3_NH_3_PbI_3_ solution processed films	Sensitivity down to 25 µC mGy^−1^ cm^−3^ Current density (photovoltaic effect) during irradiation of 25 nA cm^−2^ Current density (photoconductive effect): 7 µΑ cm^−2^	2015	^[^ ^78^ ^]^
MAPbBr_3_ single crystals	Hard X‐rays detector High *μ*_h/e_ × *τ* product of 1.4 × 10^−2^ cm^2^ V^−1^ High stability when stored Sensitivity of 0.5 µC Gy^−1^ cm^−2^ Response time of 730 µs Efficiency up to 16.4%	2016	^[^ ^89^ ^]^
MAPbBr_3_ single crystals	X‐ray detector of up to 50 keV Sensitivity of 322 µC Gy^−1^ cm^−2^ Lowest detection limit of 0.036 μGy^−1^ Deposited on Si‐substrate with the necessary circuity for optimized current transport	2017	^[^ ^90^ ^]^
Cs_2_AgBiBr_6_ single crystals	Lead‐free sensors Excellent thermal and moisture stability 30 keV X‐ray detector Sensitivity of 105 µC Gy^−1^ cm^−2^ Lowest detection limit of 59.7 nGy s^−1^	2017	^[^ ^102^ ^]^
MAPbI_3_ polycrystalline film	Large detection area of 50 × 50 cm^2^ Sensitivity of 11 µC Gy^−1^ cm^−2^ *μ*_h/e_ × *τ* product of 10^−4^ cm^2^ V^−1^	2017	^[^ ^86^ ^]^
MAPbI_3_ microcrystal wafers	38 keV X‐ray detector Sensitivity: 2.5 µC Gy^−1^ cm^−2^ *μ*_h/e_ × *τ* product of 2 × 10^−4^ cm^2^ V^−1^	2017	^[^ ^87^ ^]^
Cs_2_AgBiBr_6_ single crystals	30 keV X‐ray detector Sensitivity of 105 nC Gy^−1^ cm^−2^ Lower detection limit of 59.7 nGy s^−1^ Higher X‐ray stopping power than the hybrid organometallic systems Lead‐free system High *μ*_h/e_ × *τ* product of 2 × 10^−3^ cm^2^ V^−1^	2017	^[^ ^105^ ^]^
MAPbBr_3_ single crystals	X‐ray detector array Detection area of 3 cm × 2.8 cm × 0.7 cm 30–100 keV X‐ray detection Sensitivity of 23.6 µC Gy^−1^ cm^−2^ Response time of 26 µs	2018	^[^ ^88^ ^]^
CsPbBr_3_ nanoparticles wrapped onto rGO nanosheets	Measured X‐ray induced current of 2.8 nA Rise and fall times of ≈1 s	2018	^[^ ^94^ ^]^
CH_3_NH_3_PbI_2_Cl thin films	Electrical current density of 1.1–5.6 nA cm^−2^ (dependent on X‐ray photon energy) Rise and recovery times of 5 ms 550% higher sensitivity compared to the a‐Si X‐ray detectors	2018	^[^ ^38^ ^]^
MAPbBr_3_ single crystal	50 keV X‐ray detector operated at RT Response and recovery times of 76.2 and 199.6 µs Sensitivity of 359 µC Gy^−1^ cm^−2^	2019	^[^ ^92^ ^]^
CsPbBr_3_ quasi‐monocrystalline film	30 keV X‐ray detector Sensitivity of 55 684 µC Gy^−1^ cm^−2^ Minimum detection limit of 215 nGy s^−1^ High *μ*_h/e_ × *τ* product of 1.32 × 10^−2^ cm^2^ V^−1^	2019	^[^ ^79^ ^]^
Cs_2_AgBiBr_6_ single crystals	Sensitivity of 316 µC Gy^−1^ cm^−2^	2019	^[^ ^106^ ^]^
MAPbBr_3_ single crystals	Sensitivity of 529 µC Gy^−1^ cm^−2^ Lowest detection value of 1.21 μGy s^−1^ Photocurrent density of 2.7 nA cm^−2^	2019	^[^ ^93^ ^]^
CsPbBr_3_ solution processed single crystals	Sensitivity of 1256 µC Gy^−1^ cm^−2^ Low leakage current (0.4 nA) High *μ*_h/e_ × *τ* product of 2.5 × 10^−2^ cm^−2^ V^−1^	2020	^[^ ^98^ ^]^
Microcrystalline CsPbBr_3_ powder melt‐processed directly onto the substrate and scalable films	Sensitivity of 1450 µC Gy^−1^ cm^−2^ Film thickness of 250 µm Area of the film in cm^2^ range Hole mobility of 18 cm^2^ V^−1^ s^−1^ Lowest detectable dose rate of 500 nGy_air_ s^−1^ Theoretical limit ionization energy of 6.9 eV at 150 V	2020	^[^ ^123^ ^]^
MA_3_Bi_2_I_9_ (Seed‐crystal assisted temperature evaporation method)	Limit of detection of ≈0.62 nGy_air_ s^−1^ (out of plane) Sensitivity of ≈10 620 µC Gy_air_^−1^ cm^2^ (out of plane) Low noise signal of ≈0.006 nA cm^−2^ (out of plane operation) On storage stability demonstration for 34 days in ambient and at 60% RH High *μ*_h/e_ × *τ* product on the order of 2.8 × 10^−3^ and 1.2 × 10^−3^ cm^2^ V^−1^ for in plane and out of plane irradiation direction Dark current density of ≈0.98 nA cm^−2^ Stability under operational conditions that corresponded to ≈230 000 times of the dose required for an X‐ray chest radiograph	2020	^[^ ^116^ ^]^
Inkjet‐printed triple cation hybrid inorganic–organic semiconductor single crystal (3.7 µm thickness) Cs_0.1_(FA_0.83_MA_0.17_)_0.9_Pb(Br_0.17_I_0.83_)_3_	Flexible X‐ray sensing element comprising Au/C60/BCP/TCP/NiO*_x_*/ITO/PEN X‐ray sensitivity of 59.9 µC Gy_air_^−1^ cm^−2^ (@0.1 V) and under X‐ray irradiation intensity of 70 kVp Stable under operational (cumulative X‐Ray irradiation of 4 Gy_air_) and without encapsulation for 1 h Demonstrated flexibility and operational stability for more than 500 bending cycles (bending radius of 6 mm)	2020	^[^ ^121^ ^]^
Free seeding (solution growth) directly grown on ITO glass CsPbBr_3_ (energy bandgap 2.23 eV single crystal	X‐ray sensing element for detection and imaging applications Sensitivity of 770 µC Gy_air_^−1^ cm^−2^ (at 8 V) and under exposure to X‐ray dose of 333.69 μGy s^−1^ Dark current in the range 5–27 nA cm^−2^ High stability in ambient conditions A 4 × 4 array of X‐ray detectors was realized Dark current of the array elements at ≈10 nA cm^−2^ Current density at 40 kV X‐ray irradiation was 223 nA cm^−2^	2020	^[^ ^99^ ^]^

### 3.1 Direct X‐Ray Detectors

As already discussed, direct detection of X‐ray photons using MHP semiconductors offers an efficient, simple, and potentially inexpensive technology for numerous existing and emerging applications. To this end, detection sensitivities of 55 684 µC Gy^−1^ cm^−2^ with a low detection (LoD) limit down to 36 nGy s^−1^ have been demonstrated^[^ ^79^ ^]^ and attributed to the superior physical properties of the perovskites. Hence, the application space of such well‐performing technology is extremely broad and includes flexible and printable large‐area X‐ray imaging devices all the way to futuristic applications such as X‐ray photon energy harvesters for powering satellites in space.^[^ ^35^, ^80^ ^]^

#### 3.1.1 3D Perovskites

##### MAPbI_3_

**Figure 5:**
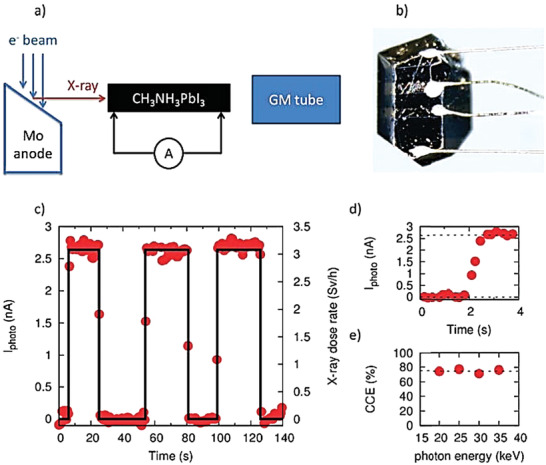
a) Schematic drawing of the X‐ray detector experimental setup. b) Photograph of CH_3_NH_3_PbI_3_ single crystal welded with gold wires. c) Temporal X‐ray response of photocurrent. d) Response speed of detector (shorter than 1 s). e) Variation of charge collection efficiency, CCE, with photon energy (≈75%). Reproduced with permission.^[^ ^81^ ^]^ Copyright 2015, American Chemical Society.

The X‐ray sensing and harvesting abilities of hybrid halide perovskites were first reported in 2015 by Náfrádi et al.^[^ ^81^ ^]^ The researchers employed the direct detection approach using MAPI single crystals as the X‐ray sensing element. The resulting devices exhibited X‐ray sensitivity and high efficiency with excellent endurance under X‐ray illumination (**Figure** **5**). The X‐ray stopping power of the MAPI single crystals was shown to be superior to Si‐based detectors; only 110 µm thick MAPI was required as compared to 1 mm thick Si to stop soft X‐rays (≈30 keV). Additionally, the sensor exhibited a high charge collection efficiency of 75 (±6)% for 20–35 keV irradiation. Another important characteristic of MAPI is that X‐ray photon absorption occurs in the bulk of the perovskite and away from the trapping/defect states that are typically present at its surface. It was argued that this is the reason why the measured X‐ray induced electrical signal did not exhibit hysteretic behavior, unlike photocurrent traces recorded under illumination with visible photons.

Náfrádi et al.^[^ ^81^ ^]^ also examined the stability of their detector against X‐ray radiation conditions, another figure of merit for practical applications. Remarkably, the photocurrent dropped by <20% under a 40 Sievert, Sv, dose (Sv is the derived unit of ionizing radiation dose in the International System of Units (SI) and is a measure of the health effect of low level of ionizing radiation on the human body). To put this number into perspective, the average yearly dose received by the International Space Station is less than 200 mSv. The authors argued that the observed degradation was caused mainly by humidity rather than radiation damage of the perovskite layer since the devices were not encapsulated. Moreover, Monte Carlo simulations highlighted the potential of MAPI single crystals as radiation shielding material, where a 1 mm thick perovskite crystal could stop 2 MeV photons.^[^ ^81^ ^]^ These results demonstrated the hardness of MAPI toward X‐ray radiation exposure and its tremendous potential for use in high‐energy radiation detectors.

As already discussed, traditional semiconductors such as amorphous Se, crystalline Si, and CdTe exhibit large photoconduction upon irradiation with X‐ray photons.^[^ ^59^, ^82^, ^83^ ^]^ The problem with traditional materials, however, is the difficulty to process/deposit them uniformly onto arbitrary substrate materials of other device components, e.g., thin‐film transistors (TFTs). This is an area where MHPs could provide important solutions due to their superb processing versatility. One example is the work by Yakunin et al.,^[^ ^78^ ^]^ which used spray‐coating to deposit 10–100 µm thick MAPI crystalline layers and build direct X‐ray detectors. The team explored two sensing strategies, one based on the photovoltaic and the second on the photoconducting effect. Photovoltaic devices with a p–i–n configuration were fabricated and used to measure the X‐ray induced charges by monitoring the build‐in potential in the device. The detectors were shown to exhibit high specific sensitivity but relatively long response times. On the other hand, the use of the photoconductive effect allowed the deployment of thicker MAPI layers, which yielded devices with improved response. However, because of the high layer thickness, a high external bias of 80 V was required to collect the generated charges efficiently. Despite the pros and cons, both the strategies demonstrated the potential of spray‐coated MAPI for the direct conversion of X‐ray photons. Notably, the reported sensitivity of 25 µC mGy_air_^−1^ cm^−3^, is comparable to that of inorganic X‐ray detectors.^[^ ^84^, ^85^ ^]^

Many of the targeted medical applications rely on large‐area X‐ray detectors that satisfy all essential figures of merit. To address this requirement, Kim et al.^[^ ^86^ ^]^ developed a polycrystalline‐based MAPI photoconductor that was argued to exhibit performance characteristics superior to inorganic X‐ray sensing elements (Table 3). The detector was manufactured onto a conventional thin‐film transistor backplane where an 830 µm thick MAPI layer was embedded between two charge transport layers composed of the polymer–perovskite mixture. The interlayers provided conformal interfaces with the conductive electrodes while simultaneously optimizing the charge extraction upon X‐ray irradiation. The choices of these hole/electron transport layers (HTL/ETL) were found to be critical in achieving high detector performance by ensuring a low dark current and efficient charge extraction. Importantly, the polycrystalline nature of the MAPI photoconductor layer addressed the technical and economic challenge associated with the use of single crystals without adversely affecting the detector performance.

**Figure 6:**
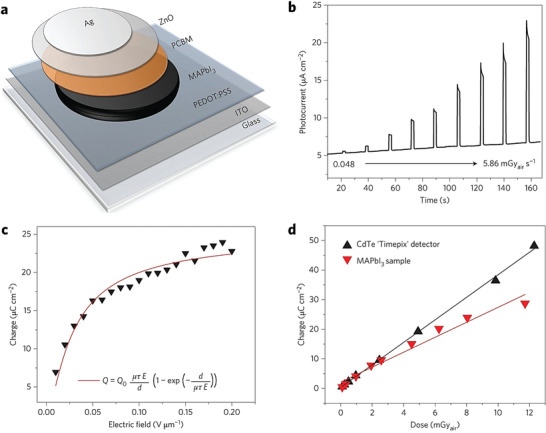
a) Schematic of the stacked MAPbI_3_ wafer‐based X‐ray detector. b) Time‐resolved photocurrent (at *E* = 0.2 V µm^−1^) as a function of dose rates. c) Charge–electric field variation at the constant dose rate, 6.72 mGy_air_ s^−1^. d) Extracted charge variation with dose rate at *E* = 0.2 V µm^−1^ of MAPbI_3_ wafer‐based device and CdTe “Timepix” reference detector. Reproduced with permission.^[^ ^87^ ^]^Copyright 2017, Springer Nature.

As already discussed, for efficient X‐ray absorption, the photoconductor layer should be approximately three times that of the material's X‐ray attenuation length. In the case of MHPs, this characteristic length is on the order of hundreds of micrometers, which represents a major technical challenge if one considers the required high structural quality of the layers.^[^ ^73^ ^]^ Shrestha et al.^[^ ^87^ ^]^ tackled this challenge by developing a room temperature mechanical sintering process to fabricate MAPI microcrystalline wafers of thickness varying from 0.2 to 1 mm. The sintering process adopted was simple and yielded polycrystalline wafers with mirror‐like reflective surfaces. The density of the resulting layers was comparable (≈3.76 g cm^−3^) to that of single crystals of MAPI (≈4.15 g cm^−3^ of the single crystal), highlighting the excellent. The 1 mm thick MAPI wafers were further tested as the active layer in a direct X‐ray detector (**Figure** **6**) using a planar inverted perovskite solar cell architecture. The devices were then irradiated with X‐ray photons with a maximum energy of 38 keV and the attenuation depth was calculated to be ≈125 µm. The estimated sensitivity of the device was 2527 µC Gy_air_^−1^ cm^−2^ at an electric field of 0.2 V µm^−1^ and the measured current showed a linear dependence on X‐ray dose. The *μ*_h/e_ × *τ* product and the ionization energy of the perovskite wafer‐based detector were comparable with those of commercial CdTe detectors.^[^ ^88^ ^]^ The low ionization energy (5 eV at 0.2 V µm^−1^) exhibited by the hybrid perovskite X‐ray detectors was attributed to: i) high collection efficiency, and ii) the low geminate charge carrier recombination rate.

##### MAPbBr_3_

**Figure 7:**
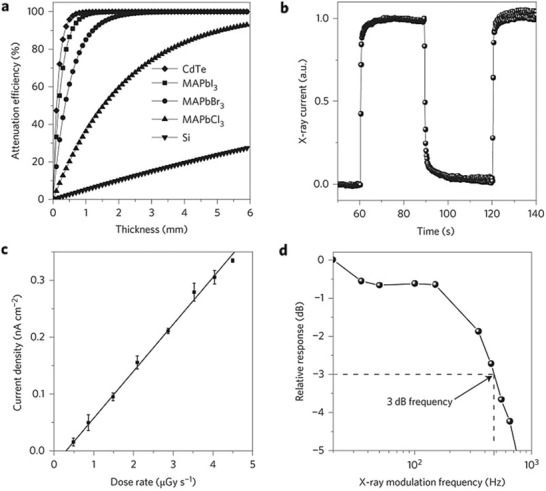
Attenuation efficiency as a function of the thickness of CdTe, MAPbI_3_, MAPbBr_3_, MAPbCl_3_, and silicon to 50 keV X‐ray photons. b) MAPbBr_3_ single‐crystal device on–off response to X‐ray illumination. c) X‐ray‐generated photocurrent at various dose rates. d) Normalized response as a function of X‐ray frequency showing that the 3 dB cut‐off frequency is 480 Hz. Reproduced with permission.^[^ ^89^ ^]^Copyright 2016, Springer Nature.

The development of efficient and inexpensive X‐ray detectors that meet the practical needs of various medical applications (e.g., cost, sensitivity, response speed) represents a critical technical challenge for most relevant material technologies. Recently, Wei et al.^[^ ^89^ ^]^ reported the development of solution‐grown, large‐area single crystals of MAPbBr_3_ that exhibited low defect density and a high *μ*_h/e_ × *τ* product. Key elements for their success was the growth of high‐quality MAPbBr_3_ crystals and the application of UV‐O_3_ treatment step to passivate the surface traps on the crystal facets. These features resulted in crystals with higher *μ*_h/e_ × *τ* product (1.4 × 10^−2^ cm^2^ V^−1^) and as such more efficient charge extraction characteristics. Interestingly, the resulting detectors exhibited an X‐ray photon stopping power higher than silicon, and sensitivities of 80 µC mGy_air_^−1^ cm^−2^; a value 10× and 70× higher than devices based on CdZnTe and MAPbBr_3_ polycrystalline films, respectively (**Figure** **7**). Furthermore, the devices showed promising stability with the shelve lifetime >2 months.

Facile integration of MHP single crystals with readout Si electronics is critical as it facilitates efficient transport of the X‐ray generated charges from the perovskite to the readout electronics. Wei et al.^[^ ^90^ ^]^ used an NH_3_Br as the terminating molecular interlayer to ensure mechanical adhesion while ensuring electrical coupling between the single‐crystal perovskite detector and the Si readout electronics beneath. The detector exhibited excellent figures of merit and was able to detect X‐ray photons with energy in the range 8–50 keV. A remarkable feature of the detector is its exceptional sensitivity, which was ≈1000× higher than commercial a‐Se detectors. Such high sensitivity detectors are particularly attractive for medical imaging applications as they can operate using a 15–20‐fold weaker dose (0.036 μGy_air_ s^−1^), which reduces the risk of exposure for the patients. In this case, the enhanced sensitivity was attributed to improved charge collection efficiency and the low noise offered by the detector‐system architecture.

X‐ray detectors that rely on sensing materials with energy‐sensitivity characteristics can enable simultaneous imaging of tissues and vessels using a one‐time radiation dose.^[^ ^91^ ^]^ Wang et al.^[^ ^88^ ^]^ developed a p–i–n (Au/poly‐TPD/MAPbBr_3_/C_60_‐doped PCBM/Ag) diode array based on a single crystal of MAPbBr_3_ 30 mm × 28 mm in size. To efficiently absorb the high energy (≈100 keV) photons, the thickness of the crystal was chosen to be ≈7 mm. The sensitivity of the detector was found to vary as a function of the photon energy, with the lower sensitivity measured at higher X‐ray photon energies. The highest sensitivity obtained was 23.6 µC mGy_air_^−1^ cm^−2^ for 30 keV at 100 V. An important observation was that the photogenerated current was found to saturate at different applied biases as the X‐ray photon energy was varied, i.e., the voltage increased with increasing photon energy. The phenomenon was attributed to carriers being generated at different depths within the perovskite crystal for different X‐ray photon energies, i.e., the higher the photon energy, the deeper the penetration depth. The result is that charges generated deeper in the crystal required higher applied fields to be detected. This interesting characteristic enabled the authors to demonstrate energy selective X‐ray imaging.

One of the main challenges for direct X‐ray detectors based on perovskite materials is the large dark current (i.e., current measured even when the detector is not exposed to high‐energy radiation).^[^ ^87^ ^]^ This is often attributed to the application of a high electric field often required to achieve high sensitivity and efficiency. Xu et al.^[^ ^92^ ^]^ developed X‐ray detectors where a high electric field could be used to increase the sensitivity of the device but without increasing the dark current. This was achieved by placing an Al electrode on top of a solution‐grown MAPbBr_3_ crystal, 13.9 mm × 13.8 mm × 2.6 mm in size, to form a Schottky contact. The latter was found to suppress the leakage current while simultaneously improving the charge collection efficiency at high applied electric fields. As a result, the X‐ray detectors showed a relatively fast response (76.2 µs) and recovery (199.6 µs) times along with good sensitivity (359 µC Gy^−1^ cm^−2^ for 50 keV at 200 V) at room temperature. Importantly, the Schottky contact‐based detectors showed higher sensitivity and a threefold faster response than the reference detector based on Ohmic gold contacts (i.e., Au/MAPbBr_3_/Au).

**Figure 8:**
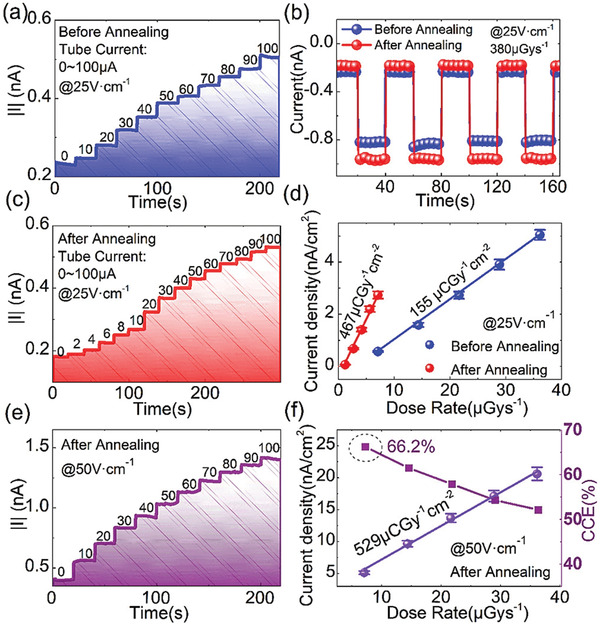
X‐ray response and sensitivity of MAPbBr_3_ as a function of time a) before and after annealing at 25 V cm^−1^. b) Annealed and unannealed device response. c)Time response of annealed sample at 25 Vcm^–1^, and d) photocurrent as a function of dose rate under 25 V cm^−1^. e) Time response of annealed sample and f) photocurrent and CCE as a function of exposure dose rate of an annealed sample under 50 V cm^−1^. Reproduced with permission.^[^ ^79^ ^]^Copyright 2019, Wiley.

In an effort to reduce the dark current, and enhance the X‐ray sensitivity of Al–ZnO (AZO)/MAPbBr_3_(crystal)/Au detectors, Li et al. exploited surface engineering to minimizing the concentration of surface trap states.^[^ ^93^ ^]^ The approach adopted is simple and relies entirely on thermal annealing to facilitate an efficient interface between AZO anode and perovskite. The detectors showed a reduced charge recombination trap density of 8.7 × 10^8^ cm^−2^ when compared to untreated devices (2.17 × 10^10^ cm^−2^), which was attributed to the filling of MA^+^ and Pb^+2^ daggling bonds with O^2−^ ions. The ensuing device showed an overall performance enhancement yielding a sensitivity of 529 µC Gy_air_^−1^ cm^−2^ at 50 V cm^−1^, a detection limit of 1.21 μGy s^−1^, photocurrent density of 2.7 nA cm^−2^ (up from 0.6 nA cm^−2^ for untreated devices) and a very low leakage current of 9 nA (at 500 V cm^−1^) (**Figure** **8**).

##### CsPbBr_3_

**Figure 9:**
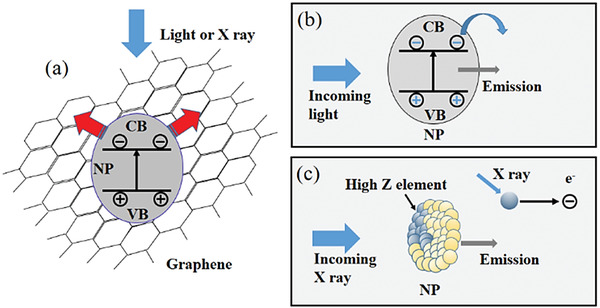
a) The schematic depiction for the conductive switching of CsPbBr_3_/rGO nanocomposites. NP: nanoparticle; CB: conduction band; VB: valence band. The response of a nanoparticle to b) visible light and c) X‐rays. Reproduced with permission.^[^ ^94^ ^]^Copyright 2018, Elsevier.

Limited temporal resolution, expensive fabrication, nonuniform sensing areas, and poor stability are some of the remaining challenges that both inorganic and hybrid MHP‐based X‐ray sensors face. To address these issues, Liu et al.^[^ ^94^ ^]^ used CsPbBr_3_ nanoparticles (CsPbBr_3_ NPs) 12 nm in diameter, to decorate reduced graphene oxide (rGO), which was the deployed as the composite X‐ray sensing element (**Figure** **9**). The combination of rGO's bipolar transporting characteristics sensitized to X‐ray photons by the CsPbBr_3_ resulted in devices with improved response times. It was argued that in this hybrid system, electrons and holes are generated within the nanoparticles upon X‐ray photon absorption and then rapidly transferred to the rGO nanosheets and ultimately to the anode and cathode. The estimated response time of the detector was ≈1 s and was found to be several times faster than the reference device based on CsPbBr_3_ NPs.

The synthesis route and processing solvents employed for the development of both the active materials and the detector systems play vital roles as they can dramatically affect the environmental impact of the studied technology. To address these issues, Wang et al.^[^ ^95^ ^]^ reported a green synthesis route able to produce in gram scale self‐assembled nanosheets (3.1 nm thick) of CsPbBr_3_. Thin solid percolating films composed of the nanosheets showed high photoluminescence yield and excellent storage stability for eight months. The CsPbBr_3_ layers, with thickness in the range of 5–25 µm, we subsequently used as the active layer in X‐ray imaging screens. An important outcome of this work was the improved understanding of the crucial role of the Pb/Cs ratio on the resulting phase of the perovskite. Crystals with the cubic phase exhibited a higher PL quantum yield (PLQY) of 68% than their orthorhombic counterparts (PLQY = 18.5%). It was argued that the green synthesis method adopted could potentially provide a route toward a commercially viable perovskite‐based X‐ray detector technology.

One of the main drawbacks of commercial X‐ray detectors based on *α*‐Se is the limited stopping power for X‐ray photons with energy >50 keV (Figure 4), while other technologies based on PbI_2_ and HgI_2_ are characterized by relatively low operational stability.^[^ ^96^ ^]^ Even though commercial systems with excellent X‐ray detection abilities do exist,^[^ ^96^, ^97^ ^]^ the high‐cost limits their widespread adoption. MHPs, such as MAPI, MAPbBr_3_, and CsAgBiBr_6_, in their single‐crystal form have proven suitable for efficient X‐ray detectors but still suffers from the relatively high density of defects, field‐induced ion migration, and poor operational stability. In an effort to address these shortcomings, Zhang et al.^[^ ^98^ ^]^ synthesized inorganic CsPbBr_3_ single crystals from solution and used them to realize X‐ray detectors. The devices comprised of Al/CsPbBr_3_/Au showed high sensitivity of 1256 µC Gy^−1^ cm^−2^ for 80 peak kilo Volt (kVp) X‐ray photons at 20 V mm^−1^, which is almost 60 times higher than commercial *α*‐Se detectors. Moreover, the combination of CsPbBr_3_ single crystals with asymmetric Al/Au electrodes was found to extend the operational lifetime of the device by suppressing field‐induced ion‐migration during operation. Using quasi‐monocrystalline layers of CsPbBr_3_ (density of 4.55 g cm^−3^, *Z* = 62.2) prepared by the hot‐pressed method, Pan et al.^[^ ^79^ ^]^ reported a record sensitivity of 55684 µC Gy_air_^−1^ cm^−2^, which is the highest among values reported for direct and indirect X‐ray detectors (see Table 3).

As already discussed, a common approach to increase the sensitivity of an X‐ray detector is to apply higher electrical bias, i.e., higher fields. Unfortunately, this simple approach often leads to increased leakage current, which in turn deteriorates the detector's performance. In an attempt to overcome this technical bottleneck, Xu et al.^[^ ^99^ ^]^ adopted a similar approach they have used for MAPbBr_3_^[^ ^92^ ^]^ and developed a Schottky‐type sandwiched photodetector comprised of Ag/CsPbBr_3_/Indium tin oxide (ITO), where the Ag/perovskite acts as the rectifying Schottky junction. The ensuing devices yielded a relatively high sensitivity value of 770 µC Gy_air_^−1^ cm^−2^ upon irradiation with a dose of 333.69 nGy s^−1^ and while reverse biased at 8 V. The improved device performance was attributed to a combination of a lower dark current (5 nA cm^−2^) and the higher quality of the crystals employed. The applicability of the proposed device concept for more complex sensor layouts was also demonstrated with the development of a functional 4 × 4 X‐ray detector array.

##### Lead‐Free Perovskites

One of the primary obstacles for the commercial deployment of hybrid lead halide perovskites in direct X‐ray detector applications is the toxicity of Pb.^[^ ^100^, ^101^ ^]^ To overcome this issue, Zhuge et al.^[^ ^102^ ^]^ used inorganic single crystals based on Cs_2_AgBiBr_6_ to detect X‐ray photons. The detectors exhibited numerous advantages including, a lead‐free composition, the inclusion of elements with high *Z*, good electrical resistivity, suppressed ionic migration (essential for preserving the crystal integrity under the application of electrical biasing), and excellent thermal and moisture stability compared to the Pb‐based perovskites. When the resulting detector was exposed to 30 keV X‐ray photons, a 4× higher sensitivity than commercially a‐Se detectors (20–105 µC Gy_air_^−1^ cm^−2^)^[^ ^103^ ^]^ was obtained. The detection limit of the Cs_2_AgBiBr_6_‐based device was also argued to be significantly lower than commercial systems. Despite the nonencapsulated nature of the sensing element, the detector demonstrated high operational stability with the detection limit of 59.7 nGy_air_ s^−1^ remaining unchanged under continuous X‐ray irradiation. The study highlighted the tremendous potential of Cs_2_AgBiBr_6_ in the field of X‐ray photon detection but also paved the path for the synthesis of other Pb‐free, and hence less toxic, materials.

From a commercial point of view, the use of Pb into a real product is strictly regulated due to its harmful effects on the health of biological systems and, more broadly, the environment.^[^ ^100^, ^101^ ^]^ Unfortunately, for X‐ray detectors, the requirement for a thick active layer/crystal makes the use of Pb‐based perovskites, particularly challenging. For example, a 2 mm thick MAPbBr_3_ crystal contains 3336 g of Pb m^−2^, which is above the safety limit that EU regulation has set as 1000 ppm.^[^ ^104^ ^]^ To overcome this issue, Pan et al.^[^ ^105^ ^]^ reported the application of an inorganic perovskite semiconductor crystal (2 mm thick), Cs_2_AgBiBr_6,_ as the active element. The resulting X‐ray detector exhibited the lowest detection limit of 59.7 nGy_air_ s^−1^, along with a low dark current (≈9.55 × 10^−16^ A Hz^−1/2^). Some of the key parameters and performance metrics of the Cs_2_AgBiBr_6_‐based X‐ray detector are summarized in Table 3.

The elimination of surface defects and shallow traps within the bulk of perovskites is essential to improve the sensitivity of X‐ray or *γ*‐ray detectors. Zhang et al.^[^ ^106^ ^]^ proposed the application of postdeposition treatment of the double inorganic perovskite Cs_2_AgBiBr_6_ crystals that relied on thermal annealing and rinsing with isopropanol. The treatment was shown reduced surface defect/trap states while neutralizing shallow electron/hole trap states. It was also found to suppress field‐driven ion migration leading to reduced leakage current levels; an essential feature for the implementation of the technology in practical sensing arrays. The improved electronic properties of the treated Cs_2_AgBiBr_6_ crystals resulted to X‐ray detectors with improved operational stability and enhanced sensitivity of 316 μGy_air_^−1^ cm^−2^ measured at 18 V bias for 39 keV X‐ray photons.

Recent effort by Zhang et al.^[^ ^107^ ^]^ focused on improving the sensitivity, stability and lower detection limit of the lead‐free inorganic metal halide direct X‐ray detectors. The absence of volatile parts, such as organic cations, and the use of Bi instead of Pb enabled the development of a highly sensitive, stable, solution‐processed and environmentally friendly X‐ray detector based on Cs_3_Bi_2_I_9_ single crystals with energy bandgap of 2.24 eV. The resulting crystals were of high quality as verified by X‐ray diffraction (XRD) measurements, while capacitance–frequency (*C*–*f*) measurements highlighted the presence of a low trap density of 1.4 × 10^10^ cm^−3^, which is comparable to 3D MHPs (10^10^–10^13^ cm^−3^)^[^ ^108^ ^]^ and significantly lower than commercial inorganic materials (10^15^–10^16^ cm^−3^).^[^ ^108^, ^109^ ^]^ Furthermore, their excellent thermal stability (up to 550 °C) and excellent stability under moisture conditions (70% RH) for more than 70 days. The engineered crystals exhibited high electrical resistivity of 2.79 × 10^10^ Ω cm, which is two orders of magnitude higher than any Pb‐based MHP detector device. This is an essential feature to reduce the dark current (≈6 pA) and thus to improve the detection limit of the device. The excellent optoelectronic properties, the superior X‐ray absorption coefficient (much higher than inorganic commercial systems, e.g., CsI and CdTe crystals) and the high responsivity of Cs_3_Bi_2_I_9_ single crystals makes them an ideal candidate for X‐ray detector applications. The X‐ray detectors comprising Au/Cs_3_Bi_2_I_9_/Au showed promising characteristics when compared to commercial detectors yielding a sensitivity value of 1652.3 µC Gy_air_^−1^ cm^−2^ (at 50 V mm^−1^), which is superior to that of a‐Se detectors (440 µC Gy_air_^−1^ cm^−2^ at 15 000 V mm^−1^), an SNR of 6.8 at a dose rate of 130 nGy_air_ s^−1^, and a minimum detection dose of 130 nGy_air_ s^−1^, i.e., a ≈42 times lower value than that required for application in medical diagnosis. The combination of low dark current and the robust operation demonstrated (thermal and operational even under X‐ray irradiation for 13 h) highlighted the potential of the Au/Cs_3_Bi_2_I_9_/Au devices for use in next‐generation direct X‐ray detectors.

#### 3.1.2 Low‐Dimensional Perovskites

From the discussion so far it becomes evident that 3D MHPs (Figure 1) face numerous challenges related to the sensitivity and operational stability of the ensuing devices^[^ ^110^ ^]^ with the most recent effort dedicated exclusively to the development of postdeposition treatment steps.^[^ ^110^ ^]^ Unfortunately, most of these strategies add to the process complexity with adverse effects on the economics of manufacturing. Recently, an attempt has been made to tackle this issue by utilizing solution‐processed 1D (see Figure 1) inorganic halide perovskite CsPbI_3_ crystals^[^ ^111^ ^]^ for X‐ray detection. Optimized detectors showed a maximum sensitivity of 2.37 mC Gy^−1^ cm^−2^, which is one order of magnitude higher than the value obtained from their 3D counterparts (**Table** **4**). On the other hand, the lowest detectable dose rate was 0.219 μCy s^−1,^ which is much lower than the minimum signal used in a regular medical diagnostics (5.5 μCy s^−1^). The reported devices also showed extremely low dark currents on the order of pA under 200 V. These exceptional performance characteristics were ascribed to an impressive *μ*_h/e_ × *τ* product, the high bulk resistivity of the material, and the improved structural stability.

**Table 4:** Summary of key performance indicators of low‐dimensional MHP‐based X‐ray detectors reported to date

Perovskite	Key performance indicators	Publication year	Refs.
2D (A_3_M_2_X_9_)–(NH_4_)_3_Bi_2_I_9_ single crystal	100 keV X‐ray detector Minimum detection limit: 55 nGy s^−1^ Anisotropic detection property: *μ*_h/e_ × *τ* product along and perpendicular to the crystal of 1.1 × 10^−2^ and 213 cm^2^ V^−1^ s^−1^, respectively High stability under exposure for 60 days	2019	^[^ ^114^ ^]^
2D Ruddlesden–Popper perovskite thin film pin X‐ray detector	Thickness of 470 nm Sensitivity: 0.276 C Gy_air_^−1^ cm^−3^ High resistivity under reverse bias 10^12^ Ω cm Lowest detected system, 5 × 10^8^ Ct s^−1^ cm^−2^ Generated *V*_OC_ ≈650 mV under X‐ray exposure Rise time of less than 500 ns and the fall time of the order of 20–60 µs	2020	^[^ ^117^ ^]^
Rb_3_Bi_2_I_9_ 2D perovskite single crystal	Record low detection limit of 8.32 nGy_air_ s^−1^ Sensitivity 159.7 µC Gy_air_^−1^ cm^−2^ *μ*_h/e_ × *τ* ≈2.51 × 10^−3^ cm^2^ V^−1^ Excellent radiation and external bias stability Excellent thermal stability with no weight loss to be observed even at high temperatures as 347 °C	2020	^[^ ^118^ ^]^
Cs_3_Bi_2_I_9_ 2D perovskite single crystal Au/Cs_3_Bi_2_I_9_/Au detector	Some cm sized single crystals High sensitivity of 1652.3 µC Gy_air_^−1^ cm^−2^ Low dark current 10 pA at 10 V external bias Low detectable limit of 130 nGy_air_ s^−1^ High operational stability under 13 h X‐ray exposure and for temperatures up to 100 °C	2020	^[^ ^107^ ^]^
1D CsPbI_3_ crystals	Sensitivity of 2.37 µC Gy^−1^ cm^−2^ High *μ*_h/e_ × *τ* product of 3.63 × 10^−3^ cm^2^ V^−1^ Low leakage current: 38 pA Lowest detection limit of 0.219 μGy s^−1^	2020	^[^ ^111^ ^]^
1D (DMEDA)BiI_5_ crystal	Sensitivity: 72.5 µC Gy^−1^ cm^−2^ 690 µm thick material can stop 93.2% of 50 keV X‐ray photons Detecting current: 2.7–8.5 nA by increasing the X‐ray dose rate (785–5499 μGy s^−1^)	2020	^[^ ^115^ ^]^
CsPbBr_3_ QDs	Bendable X‐ray detectors for 0.1–2.5 keV Detected current of 0.1–0.36 nA Sensitivity of 0.0172 mGy s^−1^ Response time of 28 ms	2019	^[^ ^35^ ^]^
0D, Pb‐free (CH_3_NH_3_)_3_Bi_2_I_9_ single crystals	Physical size 26 × 26 × 8 mm^3^ Bulk resistivity of 3.74 × 10^10^ Ω cm Highest attenuation X‐ray coefficient of 90.4% *μ*_h/e_ × *τ* product of 2.87 × 10^−3^ cm^2^ V^−1^ Sensitivity of 1947 µC Gy_air_^−1^ cm^−2^ Low dark current of 8 pA Low detection limit of 83 nGy_air_ s^−1^ Short response time of 23.3 ms Lowest baseline drift of 5.0 × 10^−10^ nA cm^−1^ s^−1^ V^−1^ High stability	2020	^[^ ^120^ ^]^

**Figure 10:**
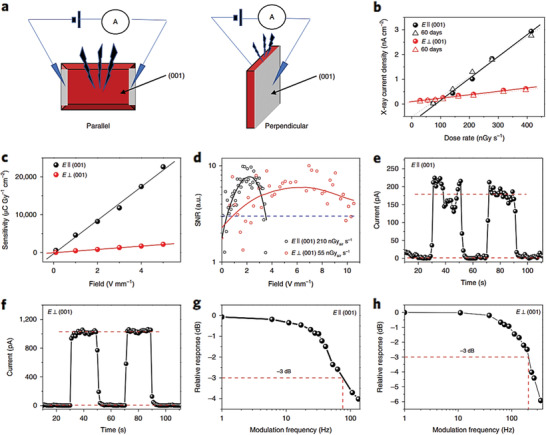
a) Diagram of parallel and perpendicular device structures. b) Anisotropic X‐ray photocurrent densities versus dose rates under pristine conditions (solid lines) and after 60 days ambient air ageing (dotted lines). c) X‐ray sensitivities and d) signal‐to‐noise ratio (SNR) of the devices in directions parallel and perpendicular to the (001) surface. The blue dotted line represents an SNR of 3. Device responses to X‐rays on tuning the X‐ray source (410 nGy_air_ s^−1^ dose rate) on and off in e) a parallel direction (1 V bias) and f) a perpendicular direction (10 V bias). Normalized response as a function of X‐ray frequency in the g) parallel and h) perpendicular directions. Reproduced with permission.^[^ ^114^ ^]^Copyright 2019, Springer Nature.

An additional concern related to 3D MHPs is their low stability due to ion‐migration occurring during device operation.^[^ ^112^ ^]^ The latter presents a major technological challenge for any practical utilization of the technology, particularly where active layers are processed via large‐area compatible techniques. The use of low‐dimensional perovskites, such as those shown in Figure 1, could address this issue as they have been shown to combine superior environmental and operational stability with low X‐ray detection threshold.^[^ ^113^, ^114^ ^]^ To this end, Zhuang et al.^[^ ^114^ ^]^ synthesized anisotropic 2D perovskite‐like materials of the form A_3_M_2_X_9_, where A = Cs, Rb, NH_4_; M = Bi, Sb; X = Br, I, and evaluated them as possible X‐ray sensing elements. Among them, single crystals of (NH_4_)_3_Bi_2_I_9_ that were grown at low temperature from solution exhibited high density (4.3 g cm^−3^), and high sensitivity with a low X‐ray detection limit of 55 nGy_air_ s^−1^. The crystals also showed suppressed ion‐migration with the ion activation energies in the perpendicular direction of 0.91 eV. Due to the anisotropy in the crystal microstructure (**Figure** **10**), the devices exhibited different X‐ray sensing characteristics depending on the crystal orientation with respect to the X‐ray irradiation direction.

In addition to the enhanced operational stability, the (NH_4_)_3_Bi_2_I_9_ crystals are nontoxic with a high X‐ray absorption coefficient.^[^ ^114^ ^]^ Specifically, a 0.99 mm thick (NH_4_)_3_Bi_2_I_9_ crystal is sufficient to stop 99% of 50 keV X‐ray photons, while MAPbBr_3_ would require a minimum thickness of 2.28 mm. Furthermore, the *μ*_h/e_ × *τ* product was found to be direction‐dependent, yielding values of 1.1 × 10^−2^ and 213 cm^2^ V^−1^ s^−1^ for the perpendicular and parallel direction to the (001) planes, respectively. The structural anisotropy was also measured by calculating charge collection efficiencies (11.4% for the parallel and 2.7% for the perpendicular). The 2D perovskite systems also showed superior thermal stability (325 and 350 K for parallel and perpendicular, respectively) and operational stability as it remained stable for >60 days.

**Figure 11:**
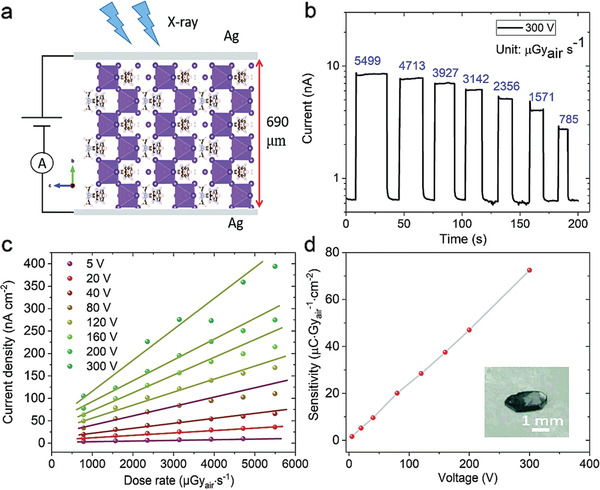
a) Schematic of (DMEDA)BiI_5_ single‐crystal X‐ray detector with a vertical structure geometry. b) Device response to X‐ray irradiation with different dose rates. c) The photocurrent density as a function of the dose rate for various bias voltages, and d) extracted detection sensitivity. Reproduced with permission.^[^ ^115^ ^]^ Copyright 2020, American Chemical Society.

In an attempt to address the Pb toxicity, Yao et al.^[^ ^115^ ^]^ proposed the use of Bi^3+^ as an alternative heavy element (*Z* = 83), which has a similar electronic configuration to Pb^2+^. The authors developed 1D systems based on (DMEDA)BiI_5_ (direct bandgap of 1.86 eV), where DMEDA is *N*,*N*′‐dimethylethanediamine‐CH_3_NH_2_CH_2_CH_2_NH_2_CH_3_^2+^. Due to its lower dimensionality and strong quantum confinement, as compared to the respective 2D and 3D structures, the new material showed lower dark current and suppressed ion‐migration when used in an X‐ray detector. Furthermore, an outstanding attenuation efficiency of 93.2% for 50 keV X‐ray photons was reported, further displaying the potential of the compound. A tunable photocurrent from 2.7 to 8.5 nA with increasing X‐ray dose has also been demonstrated (**Figure** **11**). The device sensitivity was also very high and on the order of 72.5 µC Gy^−1^ cm^−2^ at 300 V. It was argued that the calculated key performance indicators (KPIs) are superior compared to those of commercial *α*‐Se X‐ray detectors. This work highlighted the tremendous potential of this Pb‐free perovskite X‐ray detector technology while paving the way to exciting new research directions.

A recent study by Zheng et al.,^[^ ^116^ ^]^ reported on 0D Pb‐free alternative, methylammonium bismuth iodine perovskite (MA_3_Bi_2_I_9_) perovskite single crystals as an X‐ray sensing material. The authors claimed that this particular compound was able to address many of the challenges that common perovskite‐based detectors face (stability, toxicity, etc.). Specifically, the authors integrated crystals of the 0D MA_3_Bi_2_I_9_ with symmetric Au electrodes to construct X‐ray detectors that showed high‐sensitivity, low detection limit, and stable operation. The use of a large 2.5 mm thick crystal of MA_3_Bi_2_I_9_ was shown to exhibit anisotropic X‐ray sensing characteristics and several promising performance metrics including: i) a record LoD limit of 0.62 nGy_air_ s^−1^ (i.e., 20× lower than any LoD values reported to date), ii) a sensitivity value of 10 620 µC Gy_air_^−1^ cm^2^, which is comparable to best reports for 2D and 3D MHPs, and iii) stable operation under X‐ray irradiation of 23 858.5 mGy_air_ and under a high applied bias of up to 120 V. The much promising performance achieved was ascribed to different factors, including the increased activation ion energy (*E*_a_ ≈ 0.46 eV) that suppresses ion migration, hence paving the way for future studies on the development of new materials.

A simple method to improve the sensitivity of an X‐ray detector is by increasing the semiconductor's resistivity under reverse bias, i.e., suppressing leakage current. This is often achieved with the use of high quality, thick, single crystals that can withstand large applied voltages. Unfortunately, growing such large and high quality single crystals increase the cost of manufacturing of the ensuing devises and systems. To address this challenge the use of solution‐grown 2D Ruddlesden–Popper (RP) phase layered perovskite films, such as (BA)_2_(MA)_2_Pb_3_I_10_ (PbI_3_), as the X‐ray sensing element was recently proposed.^[^ ^117^ ^]^ The developed devices showed various interesting characteristics, including, low dark current (10^−9^ A cm^−2^ at zero bias), and low voltage operation (self‐powered devices). The p–i–n detectors (ITO/PTAA/(PbI_3_)/C_60_/gold) demonstrated a 10–40‐fold higher X‐ray absorption coefficient as compared to a Si detector. The existence of the internal field (generated by the different work functions electrodes employed), helped to sweep the X‐ray generated carriers to the respective electrodes, allowing the demonstration of self‐powered X‐ray detectors, which is ideal for the detection of low energy X‐ray photons. Due to the low dark current, four orders of magnitude higher signals than the dark current at zero bias were recorded – a much higher value than reference Si detectors that showed two orders of magnitude higher SNR. The high sensitivity of the detectors was attributed to the high quality of the 2D perovskite employed and the low dark current. The low dark current of the detector yielded excellent X‐ray sensitivity of 10^−5^ Gy_air_ s^−1^ for 10 keV X‐ray photons at zero bias. Irradiating the devices with X‐rays induced a signal of ≈650 mV while in the case of the reference Si detector, the resulting signal was ≈250 mV. Moreover, the X‐ray detector showed hysteresis free operation with short rise and fall times (between 1 and 10 µs). The devices also exhibited excellent stability for 30 cycles of voltage scans and under X‐ray exposure (300 s exposure time).

As already discussed, two key factors for enhancing the detection limit of perovskite direct X‐ray detectors, are the dark current, and the ion migration within the perovskite during electrical biasing (i.e., device operation). Xia et al.,^[^ ^118^ ^]^ attempted to tackle these issues by engineering single crystals of a 2D A_3_B_2_X_9_ perovskite, namely Rb_3_Bi_2_I_9,_ with an energy bandgap of 1.89 eV. The ensuing detectors (device structure: Au/Rb_3_Bi_2_I_9_/Au) exhibited excellent physical (a crystal 0.4 mm thick was enough to stop 90% of 30 keV X‐ray photons) and electronic properties (*μ*_h/e_ × *τ* = 2.51 × 10^−3^ cm^2^ V^−1^). These advantageous characteristics culminated in high sensitivity (159.7 µC Gy_air_^−1^ cm^−2^, i.e., much higher than the a‐Se detectors ≈20 µC Gy_air_^−1^ cm^−2^), high bias stability (withstand external bias up to 100 V) and a record‐low, for a direct X‐ray detector, the detection limit of 8.32 nGy_air_ s^−1^. The latter was related to the high resistivity of this perovskite crystal employed (≈2.3 × 10^9^ Ω m). Furthermore, the high ion migration energy of the Rb_3_Bi_2_I_9_ crystal (i.e., 561 meV compared to 228 meV for CsPbBr_3_) resulted in a low dark current of 114 nA at 100 V as compared to 200 nA for CsPbBr_3_. The Rb_3_Bi_2_I_9_‐based direct X‐ray detectors also showed robust operation under continuous irradiation with *γ*‐rays: the dark current increased from 1.17 to 9.53 pA while no structural deformation was observed after a total radiation dose of 480 000 Gy with 1.33 MeV *γ*‐rays.

It is well established that ion‐migration in 3D perovskite X‐ray detectors is responsible for the poor resolution, slow response, low sensitivity, and in some cases, decomposition of the functional layer. Therefore identifying strategies to minimize or even eliminate ion migration could prove critical for the success of the technology in future applications. One approach to reduce ion‐migration in 3D perovskites is to grow crystals of the highest quality possible. This is because the presence of a lower number of structural defects and the absence of grain boundaries in such crystals are known to dramatically suppress ion‐migration. The second approach that complements the first is to reduce the dimensionality of the perovskite (Figure 1), where the ion‐migration is found to be significantly lower, depending on the quality of the layers employed.^[^ ^119^ ^]^

Liu et al.^[^ ^120^ ^]^ adopted both of these approaches and demonstrated solution‐processed, 0D, Pb‐free, high‐quality MA_3_Bi_2_I_9_ single perovskite crystal of inch size for the first time. The perovskite crystals combined all features required for application in direct X‐ray detectors, including suppressed ion‐migration. The short response times (rise and fall times of ≈23.3 and ≈31.4 ms, respectively) of the vertical architecture (Au/MA_3_Bi_2_I_9_/Au) detector under X‐ray irradiation was reported. The detectors showed excellent stability under continuous operation in ambient conditions (26.5 h of continuous irradiation and exposure to ambient air 55 days), low detection limit (83 nGy_air_ s^−1^), high sensitivity, and low dark currents. Furthermore, the authors calculated the activation energies of I^−^ ions (due to weaker chemical bonding with Bi and a major contributor to ion‐migration) and showed that the lower dimensional perovskites exhibit higher values (i.e., *E*_0D_ = 1.18 eV, *E*_2D_ = 0.85 eV) as compared to the 3D perovskites (*E*_3D_ = 0.54 eV). With similar aims in mind, a printable form of the 0D inorganic CsPbBr_3_ quantum dots (QDs) was reported by Liu et al.^[^ ^35^ ^]^ The researchers demonstrated X‐ray detectors (0.1–2.5 keV) with enhanced sensitivity, improved stability and processability. Additional detector parameters, including dosage‐dependent photocurrent and operational stability, are provided in the following section.

#### 3.1.3 Large‐Area Perovskite‐Based Direct X‐Ray Detectors

For the development of unconventional X‐ray detectors, such as large‐area, flexible/conformable medical imaging devices, there is often a trade‐off between the thickness of a sensing element, its mechanical flexibility and its X‐ray stopping power. Thinner active layers tend to provide higher mechanical flexibility but the stopping power, and hence the detector's sensitivity, reduces. Mescher et al.^[^ ^121^ ^]^ attempted to address this trade‐off by using a triple cation perovskite, namely, Cs_0.1_(FA_0.83_MA_0.17_)_0.9_Pb(Br_0.17_I_0.83_)_3_, to fabricate direct X‐ray sensors via a combination of spin‐coating and inkjet‐printing techniques. Layers 3.7 µm thick were found to provide the required mechanical flexibility without compromising the performance of the X‐ray detector. The flexible X‐ray detectors exhibited various attractive features including, a sensitivity of 59.9 µC Gy_air_^−1^ cm^−2^, which is close to the highest values reported to date for triple cation perovskite sensing elements,^[^ ^122^ ^]^ and low operating voltage (0.1 V). Another attractive characteristic is the enhanced stability under X‐ray illumination and accumulative exposure of 4 Gy_air_ for 1 h without encapsulation. Finally, the sensor displays excellent mechanical flexibility even after 500 bending cycles at a bending radius of 3 mm. Overall the X‐ray detectors offered promising performance, scalable low‐temperature processing, lightweight, and mechanical flexibility, making them attractive for conformal X‐ray detector applications.

**Figure 12:**
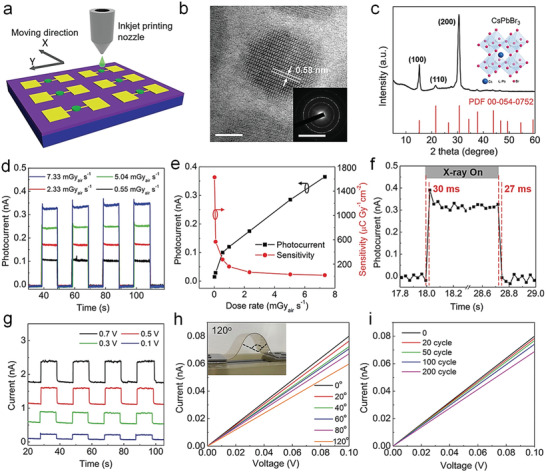
a) Schematic depicting the fabrication steps used for the development of the perovskite‐based detectors via inkjet printing. b) High‐resolution TEM of CsPbBr_3_ QDs. Scale bar: 5 nm. Inset: Selected‐area diffraction image. Scale bar: 5 nm^−1^. c) Powder X‐ray diffraction pattern of CsPbBr_3_ QDs with standard spectra. Inset: Crystal structure of CsPbBr_3_. d) ON–OFF response of the device to X‐rays under different dose rates with 0.1 V bias voltage. e) X‐ray photocurrents and sensitivity as a function of dose rate with 0.1 V bias voltage. f) Temporal response of the device under 7.33 mGy_air_ s^−1^ dose rates with 0.1 V bias voltage. g) Device response to X‐ray pulse with varying applied bias voltages under 7.33 mGy_air_ s^−1^ dose rates. h) *I*–*V* curves of the flexible device arrays at different bending angles under the X‐ray illumination of 7.33 mGy_air_ s^−1^ and 0.1 V bias voltage. Inset: Image of the real device subjected to mechanical bending. i) Representative *I*–*V* curves of the flexible device arrays after recovering from various bending cycles. Reproduced with permission.^[^ ^35^ ^]^Copyright 2019, Wiley.

The ability to process homogeneous perovskite layers that show high X‐ray sensitivity over a large‐area substrates is critical for the development of next‐generation high‐energy detectors. Unfortunately, combining the needed processing versatility with high microstructural quality of the ensuing perovskite layers, remains challenging. In an effort to address these challenges Liu et al.^[^ ^35^ ^]^ developed a printable form of 0D CsPbBr_3_ quantum dot formulations that were subsequently used to develop X‐ray detectors (**Figure** **12**a). The researchers were able to control the crystallinity of the QDs and reduce the concentration of surface defects (Figure 12b,c). The X‐ray detectors displayed exceptional operating characteristics when exposed to soft X‐rays (0.1–2.5 keV). The photogenerated current, measured at 0.1 V, could be modulated with the incident X‐ray intensity and was found to vary from 0.1 to 0.36 nA as the X‐ray dose rate increased from 0.55 to 7.33 mGy_air_ s^−1^ (Figure 12d,e). Importantly, the detector was able to detect currents down to 9 pA, which corresponded to an incident X‐ray intensity of 0.0172 mGy_air_ s^−1^, with a fast response time of 28 ms (Figure 12f).

The impact of mechanical bending on the performance characteristics of the CsPbBr_3_ QD‐based X‐ray detector (i.e., sensitivity, response times, repeatability, and stability) was also evaluated.^[^ ^35^ ^]^ The measured X‐ray induced current across the detector was found to reduce by 20% under static bending angles of 120° (Figure 12h), an effect attributed primarily to the reduced surface area exposed to the incident X‐ray photons. Overall, the X‐ray sensing array showed robust operation under 200 repeated bending cycles, which resulted in only 12% performance loss, mainly due to mechanical cracking of the sensing material (Figure 12i). The study provided an excellent demonstration of the durability, bendability, and stability of the printed perovskite X‐ray detector technology.

The processing versatility of MHPs for use in high‐energy radiation detection was extended further by Matt et al.^[^ ^123^ ^]^ The authors demonstrated a simple, scalable, and cost‐effective melt‐processing method for the deposition of CsPbBr_3_ films onto glass substrates. Their work highlighted a way to large‐area processing of efficient and low‐cost X‐ray detectors based on MHPs. A critical parameter for the success of the technique was the accurate control of the cooling rate of the layer from its melting temperature of ≈575 °C to 560 °C. To achieve this, the cooling rate was varied from 0.91 °C min^−1^ down to <0.25 °C min^−1^ with the optimum results acquired for a cooling rate of 0.125 °C min^−1^. Optimized CsPbBr_3_ films exhibited a fine structure over the whole area of the substrate. The only limiting factor regarding the scalability of the layer deposition was the size of the substrate. The resulting CsPbBr_3_ layers exhibited a specific resistance of 8.5 × 10^9^ Ω; 250 µm thick films showed similar specific resistance to 1 mm thick layers. As‐fabricated detectors were exposed to a 50 Hz X‐ray source operated at 70 kVp. The sensitivity of the detectors was evaluated, yielding a value of 1450 µC Gy_air_^−1^ at 300 V; a value comparable to state‐of‐the‐art Cd(Zn)Te X‐ray detectors, and superior to *α*‐Si X‐ray detectors. The LoD limit was also calculated yielding 500 nGy_air_^−1^, which satisfies the requirements for medical applications.

Recently Gill et al.^[^ ^38^ ^]^ demonstrated a low cost, easy to fabricate, sensitive, and flexible X‐ray sensing element using a planar inverted perovskite device architecture. The CH_3_NH_3_PbI_2_Cl‐based X‐ray sensing layer exhibited reversible, stable, and fast sensing behavior when exposed to X‐ray photons with energy varied from 60 to 150 kVp. The particular materials combination enabled efficient dissociation of the X‐ray generated electron–hole pairs and their subsequent collection at the respective electrodes. The detected electrical signal was found to vary linearly with the X‐ray dose from 1.1 to 5.6 nA cm^−2^ for 60–150 kVp, respectively. The best performance was achieved in devices incorporating a 270 nm thick perovskite layer. The extracted rise and the recovery times were also measured yielding values ≈5 ms. Overall, the demonstrated CH_3_NH_3_PbI_2_Cl X‐ray detector exhibited 550% higher sensitivity than *α*‐Si reference detector when exposed to X‐ray photons with the same energies.

Another important characteristic of MHP‐based direct X‐ray detectors is their spatial configuration. In 2014 Oh et al.^[^ ^83^ ^]^ developed the first single‐pixel detector based on polycrystalline MAPbI_3_, that could be scanned across a *x*–*y* plane and ultimately record 2D X‐ray images. Although the scanning time required to record a 2D image was long, the work motivated the scientific community to develop the first MHP‐based X‐ray detector arrays. Soon enough, the first linear detector array (LDA) based on MHP sensing materials was demonstrated by Wei et al.^[^ ^90^ ^]^ featuring 200 µm large pixels. The technology was further improved with the development of 2D arrays^[^ ^86^ ^]^ that were able to deliver faster imaging times with better spatial resolution, both very appealing for flat‐panel X‐ray imaging applications. Importantly, the deposition techniques used to fabricate the 2D imaging arrays were similar to those used for printable photovoltaics, namely, doctor blade and spray coating. The low‐temperature processing characteristics of MHPs allowed their deposition directly onto temperature‐sensitive readout electronics, further simplifying the overall manufacturing of the detector arrays. **Table** **5** summarizes some of the advantageous characteristics associated with perovskite‐based direct X‐ray detectors when compared to incumbent technologies.

**Table 5:** List of potentially advantageous characteristics associated with MHP‐based direct X‐ray detectors reported to date over commercial technologies

Potentially lower manufacturing cost (materials, processing, and system integration). Large‐area processing on arbitrary substrate materials including various plastics. Smaller amount of active materials required due to high stopping power. Higher X‐ray attenuation coefficients than numerous commercial technologies. Potential for more environmentally friendly (less toxic, recyclable, etc.) materials and systems.

### 3.2 Indirect Metal Halide Perovskite X‐Ray Detectors

The majority of the commercially available large‐area indirect X‐ray detectors, rely on the use of scintillating elements.^[^ ^124^ ^]^ The role of a scintillator is to convert ionizing radiation, such as X‐rays and *γ*‐rays, into visible photons with high quantum efficiency. The fabrication of conventional inorganic scintillators involves the high‐temperature (up to 1850 °C) sintering of crystalline solids into desired shape and form, which when comes down to device integration, poses significant manufacturing challenges.^[^ ^124^ ^]^ This is why major recent effort has focused on developing scintillators that are simpler to manufacture over large‐area substrates using high throughput deposition techniques at significantly lower temperatures.

As already discussed extensively in the previous section, solution‐processable MHPs have demonstrated tremendous potential for the direct detection of X‐rays and *γ*‐rays, but their responsivities at photon energies above 10 keV remain relatively low. Increasing the sensing layer thickness has shown to improve the responsivity, but unfortunately, the latter approach limits the carrier diffusion length leading to inefficient extraction. This challenge can, in principle, be addressed using X‐ray scintillators. The KPIs of a scintillator includes:

1) Short decay time.
2) High LY.
3) High detection efficiency.
4) Emission of visible photons.

To date, perovskite‐based X‐ray scintillators^[^ ^125^, ^126^ ^]^ with minimum decay time as short as 0.7 ns, already significantly shorter than those of established inorganic systems such as LSO(Ce) (≈41 ns), high LY (24 photons MeV^−1^), and high conversion efficiencies (49%) have been reported. This level of performance has been reached through careful crystal engineering of the perovskite layer and the elimination of defects even in millimeter thick films. However, considering the versatile chemistry of MHPs and the different material dimensionalities accessible, one could argue that further improvements are certainly possible.

**Figure 13:**
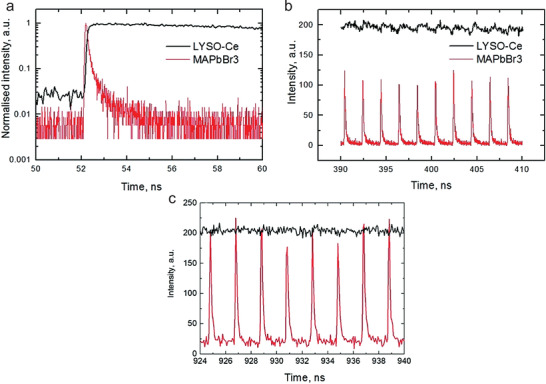
a) Normalized scintillation decay curves observed upon excitation with 14 keV X‐ray pulses in MAPbBr_3_ (*T* = 77 K, red curve) in comparison with LYSO‐Ce (*T* = 292 K, black curve). b) The sequence of X‐ray pulses from electron bunches in the synchrotron ring (time interval 2 ns, FWHM = 60 ps) as recorded with an ID100 photon counter using MAPbBr_3_ (red) and LYSO‐Ce (black) at *T* = 77 K. Reproduced with permission.^[^ ^127^ ^]^Copyright 2019, Royal Society of Chemistry.

Achieving a time resolution below 10 ps requires simultaneous optimization of the light generation within the scintillator, and its transmission and coupling to the photodetector. Time resolutions of 10 ps require scintillator materials with LY at ≥140 000 photons MeV^−1^ with <1 ns response times. Mykhaylyk et al.^[^ ^127^ ^]^ managed to develop a MAPbBr_3_‐based X‐ray scintillator (14 keV) that operates in the temperature range 50–130 K with LY of 90 000 at 77 K and response time of 1 ns (**Figure** **13**). Most importantly, the work highlighted the possibility of operating the scintillator above 50 K.

**Figure 14:**
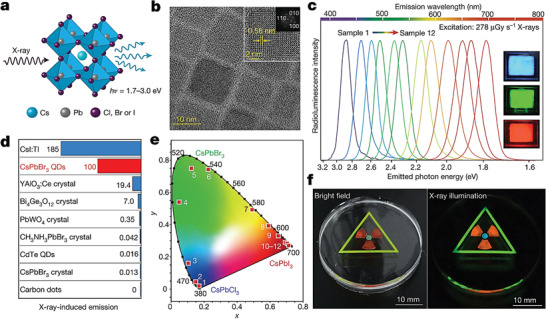
a) Schematic representation of X‐ray interaction with an all‐inorganic perovskite lattice. b) Low‐resolution transmission electron microscopy (TEM) image of the as‐synthesized CsPbBr_3_ nanocrystals. The inset shows a high‐resolution TEM image along the [100] zoom axis. c) Tunable luminescence spectra of the perovskite QDs under an X‐ray dose rate of 278 μGy s^−1^ at a voltage of 50 kV. The insets show photographs of the thin‐film samples, which emit blue, green, and red colors, respectively, upon X‐ray irradiation. d) Comparison of the optical sensitivity of various scintillator materials for X‐rays produced at 10 kV. e) Commission Internationale de l'Eclairage (CIE) chromaticity coordinates of the X‐ray‐induced visible emissions measured for the various samples. f) Multicolor X‐ray scintillation (left, bright‐field imaging; right, X‐ray illumination at a voltage of 50 kV) from three types of perovskite nanocrystal scintillators. Reproduced with permission.^[^ ^126^ ^]^Copyright 2018, Springer Nature.

Chen et al.^[^ ^126^ ^]^ went a step further by taking advantage of the flexible chemistry and tunable dimensionality of the perovskite materials, and demonstrated flexible, color‐tunable perovskite scintillators based on nanocrystals (≈9.6 nm in size) (**Figure** **14**). Higher sensitivity, as compared to that of high‐efficiency bulk CsI:Tl scintillator, high emission yield, low detection limit (13 nGy s^−1^, which is 400× lower than typical imaging doses), and fast scintillation times (4.6 ns) are some of the key parameters extracted from the developed devices. Moreover, the tunability of the X‐ray induced emission is attractive for use in multicolor X‐ray imaging applications. Overall, the work addressed various technical challenges facing incumbent scintillator technologies including, manufacturability (i.e., cost) and performance.

Commercially indirect X‐ray scintillator systems suffer from a number of disadvantages, including high cost due to complex fabrication, and incompatibility with flexible X‐ray imaging applications.^[^ ^128^ ^]^ Additional technical limitations include long response times. Heo et al.^[^ ^129^ ^]^ developed a cost‐effective, high response, high spatial resolution, and stable CsPbBr_3_ nanocrystal‐based scintillator element. High PL quantum yield (95% at 550 nm), short PL decay time (2.87 ns), the fast response time (120 ns), higher absorption, conversion efficiencies and spatial resolution (9.8 lp mm^−1^) are the key figures of merit. The detailed figures of merit of the CsPbBr_3_ scintillator are summarized in **Table** **6**.

**Table 6:** Summary of key performance indicators of MHP‐based X‐ray scintillators reported to date

Perovskite	Key performance indicators	Publication year	Refs.
C_3_PbI_4_ and C_3_PbBr_4_ nanocrystals	Decay time of 0.7 ns Light emission at 558 nm	2004	^[^ ^132^ ^]^
PhE‐PbBr_4_ single crystal	Decay time of 9.9 ns Light emission at 440 nm Time resolution of 0.7 ns LY of 24 Detection efficiency of 24% at 67 keV	2008	^[^ ^133^ ^]^
2D (EDBE)PbCl_4_ crystal	Response time of 7.9 ns Light emission at 520 nm PL efficiency of 120 000 photons MeV^−1^	2016	^[^ ^142^ ^]^
CsPbX_3_ (X = Cl, Br, I) QDs	Low detection limit of 13 nGy s^−1^ Response time of 44.6 ns	2018	^[^ ^126^ ^]^
CsPbBr_3_ nanocrystals	Response time of 120 ns Spatial resolution of 9.1 lp mm^−1^ PLQY of 95% Light emission at 550 nm Decay time of 2.8 ns	2018	^[^ ^129^ ^]^
Inorganic and hybrid organometallic perovskite crystals	Determination of the optimum temperature that scintillators should operate; operation at <50 K is suggested for all types	2018	^[^ ^130^ ^]^
CsPbBr_3_ self‐assembled nanosheets	LY of 63% High stability with an LY = 94% maintained after eight months storage	2019	^[^ ^95^ ^]^
MAPbBr_3_ crystals	Detection of 14 keV X‐rays Time resolution of 10 ps Response time of 1 ns Light emission at 560 nm LY of 90 000 at 77 K	2019	^[^ ^127^ ^]^
MAPbCl_3_ single crystal	50 keV X‐ray scintillator Light emission at 432 nm Low detection limit of 114.7 nGy s^−1^	2020	^[^ ^92^ ^]^
(C_8_H_17_NH_3_)_2_SnBr_4_ onto a polymethyl methacrylate (PMMA) film	Light emission at 596 nm with PL decay time of 3.34 µs Image resolution of 200 µm Lowest detectable signal of 104.23 μGy s^−1^ (for 40 kV X‐rays) Mechanical flexibility and operational stability under X‐ray irradiation	2020	^[^ ^134^ ^]^

Xie et al.^[^ ^130^ ^]^ studied the temperature and dose‐dependent X‐ray luminescence in various perovskite single‐crystals, including CsPbBr_3_. The study paved the way to an improved understanding of the relevant processes for different hybrid and inorganic metal halide perovskite materials with emphasis on the importance of the operating temperature as it is critical for high‐resolution imaging. Furthermore, the authors showed that the type of halogen employed (Cl, Br, I) play an important role not only on the energy bandgap of the formed perovskite but also on several other parameters including i) the threshold of the quenching temperature for X‐ray luminescence (for MAPbCl_3_ measured at 100 K the linewidth of the signal narrows to 1 nm, while for MAPbI_3_ at 60 K it reduces to 10 nm), ii) the rate of quenching, and iii) the intensity of the X‐ray luminescence signal. The effect of the X‐ray dose to the measured signal linewidth measured at 10 K was found to vary for the different perovskite crystals tested. Even for the inorganic perovskites, there is a threshold temperature (100 K) below which the line width of the emitted signal narrows from 23 down to 2 nm and even to 1.5 nm below 40 K. The X‐ray luminescence linewidth remains unaffected by the dose when measured at a constant temperature of 10 K. Finally, the doping of MAPbBr_3_ with Bi^3+^ in various concentrations helped to increase the strength of the thermal quenching and enhanced the stability of the scintillating material in the air compared to the undoped systems.

**Figure 15:**
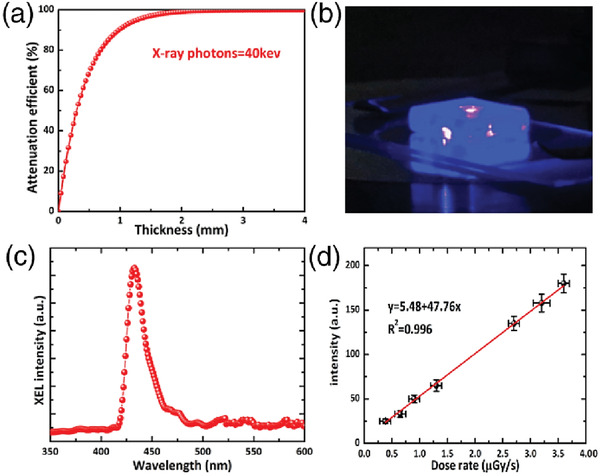
a) Attenuation efficiency of MAPbCl_3_ SCs at various thicknesses. b) Optical image of MAPbCl_3_ SCs grown on a glass substrate with color scientific grade CCDs excited with 266 nm laser. c) XEL spectra of MAPbCl_3_ SCs. XEL spectra excited by an X‐ray from a tungsten cathode at room temperature (80 kV, 4 mA). d) Integrated XEL of MAPbCl_3_ SCs as a function of the dose rate. Reproduced with permission.^[^ ^92^ ^]^Copyright 2020, The Optical Society.

Reducing the detection limit of the detector could lead to reduced X‐ray dose rates, important for the health and safety of the users and/or patients, and improved image contrast. Recently Xu et al.^[^ ^92^ ^]^ developed a low cost, solution‐processed MAPbCl_3_ single‐crystal (10 × 10 × 3.5 mm^3^) scintillator system with outstanding performance characteristics (**Figure** **15**). The scintillator showed a low detection limit of 114.7 nGy s^−1^ at 50 keV X‐rays, a feature attributed to the high X‐ray absorption coefficient of the MAPbCl_3_ crystal and was shown to be comparable to commercial scintillators based on NaI and CsI.^[^ ^131^ ^]^ The relative LY of the scintillator measured at 432 nm increased linearly with increasing dose rate, a highly desirable feature for practical applications.

The concept of “quantum scintillator” was introduced in 2004 by Shibuya et al.^[^ ^132^ ^]^ to describe a direct bandgap perovskite semiconductor in which recombination of excitons through defect states could be avoided resulting in ultrafast and highly efficient scintillation even at room temperature. This was achieved using the natural multiple quantum well structure of the lead‐halide‐based perovskite (n‐C_6_H_13_NH_3_)_2_PbI_4_, abbreviated to CmPbX_4_. For the C_3_PbBr_4_ compound, the team demonstrated decay times of 2.8 ns, whereas C_6_PbI_4_ crystals exhibited even shorter decay times of 0.7 ns. These values are by far superior to any inorganic scintillating materials (e.g., NaI(Tl) ≈230 ns, BGO ≈60 ns, and LSO(Ce) ≈41 ns). In addition to the fast response, the perovskite “quantum scintillators” emitted visible light (558 nm), which can be seen as an added advantage when compared to other technologies such as BaF_2_ scintillators which emit in the UV region.

In an effort to address the low X‐ray detection efficiencies of the aforementioned CmPbX_4_ perovskites at photon energies >20 keV, Kishimoto et al.^[^ ^133^ ^]^ exploited a different “quantum scintillator” material, namely, bis(phenethylammonium) tetrabromoplumbate (PhE‐PbBr_4_), and successfully demonstrated X‐ray scintillation with a short decay signal of 9.9 ns, a high LY (22 ± 2 photons MeV^−1^) and relatively high detection efficiency (24%) at 67.4 keV. Using this system, the team was able to successfully record the decaying *γ*‐rays emitted from ^61^Ni with a minimum time resolution of 0.7 ns. These early studies highlighted the tremendous potential of perovskites as novel X‐ray scintillating materials, even at higher photon energies.

**Figure 16:**
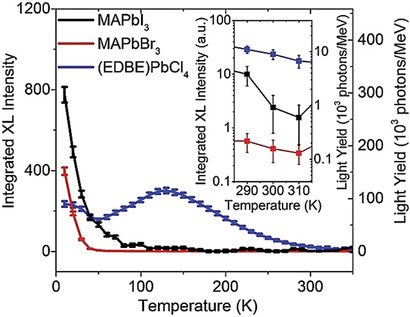
Temperature dependence of the light yields. Light yields of MAPbI_3_, MAPbBr_3_, and (EDBE)PbCl_4_ obtained from the integrated X‐ray excited luminescence intensities at various temperatures, from 10 to 350 K. Reproduced with permission.^[^ ^125^ ^]^Copyright 2016, Springer Nature.

From the discussion so far, it becomes evident that reducing the dimensionality of the scintillating materials benefits the photoluminescence efficiency during scintillation. To this end, Birowosuto et al.^[^ ^125^ ^]^ showed that the large exciton binding energy that exists in 2D perovskites could suppress losses of the detected optical signal due to thermal quenching. Although both 2D and 3D perovskite‐based scintillators generated X‐ray induced luminescence yields of 120 000 photons MeV^−1^ at low temperatures, at room temperature the large exciton binding energy of the 2D material (i.e., (EDBE)PbCl_4_) was found to reduce thermal effects compared to 3D perovskites, and a moderate light yield of 9000 photons MeV^−1^ was maintained (**Figure** **16**). On the other hand, scintillators based on the 3D perovskites MAPbBr_3_ and MAPbI_3_ exhibited significantly smaller room temperature LY of <1000 photons MeV^−1^.

Along the same lines, Cao et al. developed an environmentally friendly and cost‐efficient organic–inorganic 2D perovskite (C_8_H_17_NH_3_)_2_SnBr_4_ film embedded into PMMA film.^[^ ^134^ ^]^ The developed material demonstrated an excellent absolute PLQY of up to 98%, with long‐lived PL signal (3.34 µs at 596 nm) under X‐ray irradiation. Moreover, the sensitivity achieved was in line with medical safety standards with the emission threshold at 104.23 μGy s^−1^. Regarding the biological toxicity of (C_8_H_17_NH_3_)_2_SnBr_4_, it was argued to be lower than CsPbBr_3_‐based scintillators due to the absence of Pb. The detection setup was completed with the use of a low‐cost CCD camera, which allowed image resolutions down to 200 µm. Furthermore, the incorporation of PMMA provided the composite scintillator with desirable mechanical flexibility without compromising its stability under continuous X‐ray irradiation.

The fabrication of high PLQY, solution‐processed materials for X‐ray scintillators is always a challenge. Embedding perovskite quantum dots inside a 3D perovskite (QD‐in‐P) where the perovskite acts as the host and the QDs as the light‐emitting centers is one of the approaches explored recently.^[^ ^135^ ^]^ However, in such systems, the existence of a large Stokes shift is essential in order to avoid self‐absorption within the relatively thick scintillating layer. The solution proposed composed of CsPbBr_3_:Cs_4_PbBr_6_ composite blend where Cs_4_PbBr_6_ was the large bandgap host material and the CsPbBr_3_ was the light‐emitting quantum dots.^[^ ^135^ ^]^ The Type‐I heterostructure formed between the bulk Cs_4_PbBr_6_ and the CsPbBr_3_:Cs_4_PbBr_6_ QDs was shown to be an efficient scintillator. The advantages of perovskite indirect X‐ray detectors over commercially available technology have been listed in **Table** **7**.

**Table 7:** Advantageous characteristics of perovskite‐based indirect X‐ray detectors/scintillators as compared to commercially available material technologies

Lower manufacturing cost. Compatibility with flexible substrates due to low‐temperature processing requirements. Higher X‐ray coefficients as compared to Se‐, TlBr‐, and CdTe‐based detectors. Shorter response times than various commercially available technologies. Higher sensitivities compared to CsI:Tl scintillator systems. Low detection limits as compared to NaI and CsI. Faster response times than numerous commercial technologies. Emission in the visible part of the electromagnetic spectrum.

### 3.3 Metal Halide Perovskites in Gamma‐Ray Detectors

#### 3.3.1 Direct Gamma‐Ray Detectors

**Figure 17:**
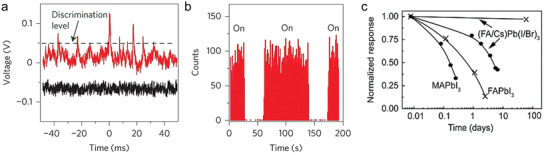
a) Signals measured from a MAPbI_3_ perovskite detector while been exposed to a low‐activity ^137^Cs source (red) and under no exposure (black). The curves are offset for clarity. b) Signal‐to‐background comparison for the MAPbI_3_ perovskite detector with and without exposure to ^137^Cs source (2.2 MBq, manual placement). c) Operational stability (crosses) and storage stability (circles) of detectors based on Cs_0.1_FA_0.9_PbI_2.8_Br_0.2_ MAPbI_3_ and FAPbI_3_. a,b) Reproduced with permission.^[^ ^136^ ^]^ Copyright 2016, Springer Nature. c) Reproduced with permission.^[^ ^137^ ^]^Copyright 2017, Springer Nature.

The application of metal halide perovskites for the direct detection of *γ*‐rays (i.e., gamma photons; Figure 2) was first reported by Yakunin et al.^[^ ^136^ ^]^ in 2016. The researchers exploited the physical and electrical properties of various mixed MHPs (MAPbI_3_, FAPbI_3_, and iodine treated MAPbBr_3_, where MA is methylammonium and FA is formamidinium) to demonstrate *γ*‐ray detection in solid‐state devices and compare their performance to commercial detector material technologies such as CdTe and CdZnTe. The single‐crystal perovskite devices demonstrate an overall efficiency of current to charges of 19% when exposed to different radioactive sources (^11^C and ^137^Cs) that emitted *γ*‐rays (0.96 MeV) with intensity as low as 2.2 MBq. Importantly, the detectors demonstrated the potential for single *γ*‐photon counting, an extremely challenging task (**Figure** **17**a,b). High stability of over eight months, while being subjected to intermittent use as a *γ*‐ray detector, was demonstrated and attributed to the low surface‐to‐volume ratio of the large single crystals as compared to polycrystalline films or nanostructured perovskite‐based detectors. A noteworthy observation was the superior performance of FAPbI_3_ single crystals as compared to similar size MAPbI_3_ crystals. In particular, FAPbI_3_‐based detectors showed greater operational stability at higher applied voltages, lower dark current (low noise), and 100× higher counting rates, clearly highlighting the potential of the technology for energy‐resolved spectroscopy.

The development of high‐resolution energy spectra *γ*‐ray detector using MHPs requires a high *μ*_h/e_ × *τ* × *E* product, where *E* denotes the applied external electric field. Although the application of higher *E* increases the drift velocity of the photogenerated charge carriers, it also enhances both the dark current/noise and ion‐migration within the perovskite. Wei et al.^[^ ^77^ ^]^ managed to overcome this limitation and demonstrated low operation field (1.8 V mm^−1^) *γ*‐ray detectors able to resolve the energy spectrum of ^137^Cs at room temperature. The researchers achieved this by increasing the bulk resistivity of the single crystal (3.6 × 10^9^ Ω cm) using large‐size dopants (compensated CH_3_NH_3_PbBr_2.94_Cl_0.06_ single‐crystal alloy instead of CH_3_NH_3_PbBr_3_ or CH_3_NH_3_PbCl_3_), and by implementing a guard ring electrode architecture. The dopant‐compensated CH_3_NH_3_PbBr_2.94_Cl_0.06_ crystals showed enhanced hole and electron mobilities of 560 and 320 cm^2^ V^−1^ s^−1^, respectively, yielding a significantly higher *μ*_h/e_ × *τ* product. Because of these improvements, the charge collection efficiency (CCE) of the CH_3_NH_3_PbBr_2.94_Cl_0.06_‐based detectors was considerably higher than devices based on pristine CH_3_NH_3_PbBr_3_ and CH_3_NH_3_PbCl_3_ crystals.

Another major pitfall of most MHPs is their poor environmental stability.^[^ ^112^ ^]^ In the case of single crystals, this instability often manifests in phase transformation from cubic to hexagonal within 24 h following the crystal growth. Therefore, addressing this major pitfall is critical for commercial exploitation of the technology in *γ*‐ray detectors. Nazarenko et al.^[^ ^137^ ^]^ fabricated stable single crystals of Cs*_x_*FA_1−_ *_x_*PbI_3−_ *_y_*Br*_y_* (*x* = 0–0.1, *y* = 0–0.6) with various thicknesses between 0.2 and 15 mm and used them for direct detection of *γ*‐ray with energies in the range 0.02–1 MeV. A critical development was the replacement of 10% of the FA ions with Cs. The resulting crystals exhibited much higher stability with signs of detector degradation appearing only after 20 days of storage. The detector stability was extended further to two months by replacing I ions with Br (*y* = 0.2–0.4). The higher chemical stability of the crystal was accompanied by improved operational stability of the device as compared to reference detectors based on FAPbI_3_ (Figure 17c).

**Figure 18:**
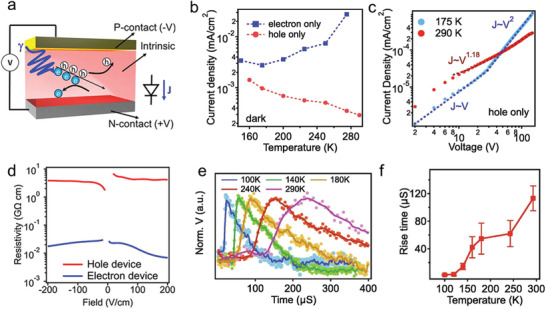
a) Schematic of *γ*‐ray detector structure and charge ionization process. b) Current density values of E‐only (blue) and H‐only (red) devices at 180 V as a function of temperature. c) Log–log plot of the H‐only device in the dark at 290 and 175 K, linear fittings represented with dotted lines. d) Measured dark resistivity of the H‐only device (red) and E‐only device (blue). e) Typical pulses at various temperatures and 20 V bias under ^137^Cs source. The pulses are shifted horizontally to show the rising edge. f) Averaged rise time as a function of temperature. Adapted with permission.^[^ ^138^ ^]^Copyright 2020, Elsevier.

Low‐cost combined with high energy resolution, high SNR and room temperature operation are key characteristics of *γ*‐ray detectors required for a variety of applications in astrophysics, medicine, nuclear material detection, etc. MHP‐based *γ*‐ray detectors appear to satisfy many of those requirements offering room temperature operation while combining high energy resolution (<10%) with low dark current even under high electric fields. Despite the promising early work, however, several issues remain. For example, different MHPs exhibit different energy detection spectra, with inorganic systems such as the CsPbBr_3_, known to detect a broad range from 32.3 to 662 keV, whereas hybrid systems (e.g., MAPbBr_3−_ *_x_*Cl*_x_*) are known to detect only high energy *γ*‐photos (0.1–10 MeV).^[^ ^77^ ^]^ The impact of operating temperature on the reaction times of the detector is also known to vary. To address these challenges, Liu et al.^[^ ^138^ ^]^ proposed the use of a unipolar p–i–p device architecture (H‐device) based on MAPbBr_3−_ *_x_*Cl*_x_* as the *γ*‐ray detector, employing suitable electrodes to lower the dark current (**Figure** **18**). The dark resistivity of the device was found to reduce as the applied bias increased, hence limiting the maximum applied electric field to 50 V cm^−1^. At higher electric fields, the dark current dominates, making *γ*‐ray detection impossible.

The same group also showed that under *γ*‐ray irradiation, the hole generated current increased with lowering the temperature.^[^ ^138^ ^]^ The opposite was found to be true for p–i–n devices. The behavior was attributed to different charge transport mechanisms for holes and electrons in the different device architectures. The high resistivity of the H‐device to dark current allowed device operation under high external electric fields up to 400 V cm^−1^ yielding significantly larger signals than the corresponding p–i–n detectors. Lowering the temperature of the p–i–p detector was also shown to reduce the response time more than increasing the applied electric field.

Compositional engineering of halides is another method that can be used to improve the physical and electronic properties of MHPs, including their *γ*‐ray detection efficiency. Rybin et al.^[^ ^139^ ^]^ showed that incorporating Cl (up to 10% concentration) into MAPbBr_3_ single crystal (2 mm long) suppresses the Br ion migration, ultimately resulting in lower dark current and balanced electron and hole mobilities. The optimized MAPbBr_3−_ *_x_*Cl*_x_* crystal‐based detectors showed higher resistivity that resulted to *γ*‐ray spectral resolution of 35%. A summary of the performance parameters of recently reported MHP‐based *γ*‐ray detectors is given in **Table** **8**.

**Table 8:** Summary of key performance indicators of MHP‐based *γ*‐ ray detectors reported to date

Perovskite	Key performance indicators	Publication year	Refs.
MAPbI_3_ and FAPbI_3_ single crystals	Room temperature operation *μ*_h/e_ × *τ* product of 10^−2^ cm^2^ V^−1^ Overall efficiency of 19% *γ*‐Ray detector (0.96 MeV) Detected intensity of 2.2 MBq	2016	^[^ ^136^ ^]^
Solution processed CH_3_NH_3_PbBr_3−_ *_x_*Cl*_x_* single crystal	Room temperature operation *μ*_h/e_ × *τ* product of 1.8 × 10^−2^ cm^2^ V^−1^ 10 times lower dark current compared to MAPbBr_3_ single crystal‐based systems Excellent stability after one month storage under ambient conditions as well as under operation	2017	^[^ ^77^ ^]^
Cs*_x_*FA_1−_ *_x_*PbI_3−_ *_y_*Br*_y_* (*x* = 0–0.1, *y* = 0–0.6) single crystals	*μ*_h/e_ × *τ* product of 0.12 cm^2^ V^−1^ Extended stability for over two months shelve storage	2017	^[^ ^137^ ^]^
Organic–inorganic perovskite layered type compound (C_6_H_5_C_2_H_4_NH_3_)_2_PbX_4_	*γ*‐Ray scintillator system LY of 14 000 photons MeV^−1^ *γ*‐Ray detected: 122–662 keV Short decay time of 11 ns	2017	^[^ ^141^ ^]^
MAPbBr_3−_ *_x_*Cl*_x_* single crystals under p–i–p architecture	Size in the range 1–5 mm in length, 1–3 cm in thickness Dark resistivity of 1 × 10^9^ Ω m under high bias Operating at biases of up to 400 V cm^−1^ Operating at 260 K Efficient *γ*‐ray detection with high signal to noise ratio at 150 V cm^−1^ Operation for few hours under constant biasing Rise time of 46 µs at 100 V at RT Rise time of 10 µs at 100 V at 100 K	2020	^[^ ^138^ ^]^
MAPbBr_3−_ *_x_*Cl*_x_* single crystals Cr/MAPbBr_2.85_Cl_0.15_/Cr setup	Crystal size of ≈2 mm Room temperature operation Spectral resolution of 35% at 100 V for 59.6 keV *γ*‐rays from ^241^Am No hysteresis and high resistivity of 1.522 GΩ cm High operational stability for a period of 8–12 weeks	2020	^[^ ^139^ ^]^

#### 3.3.2 Indirect Gamma‐Ray Detectors

Time‐of‐flight (TOF) positron emission tomography (PET) is an essential medical imaging technique that relies heavily on *γ*‐ray detectors. Current TOF‐PET imagers employ fast *γ*‐ray photon scintillators and photodetectors. Important characteristics of the *γ*‐ray detector used in this type of application include high photon yield, high energy resolution, high radiation hardness, fast response and recovery times, and chemical/environmental stability. Inorganic *γ*‐ray scintillating materials such as GSO:Ce and LSO:Ge satisfy several of these requirements but often suffer from long decay times (≈40 ns). To this end, hybrid materials^[^ ^50^, ^138^, ^140^ ^]^ offer short decay times but are generally unstable with a low material density (1 g cm^−3^). Thus, the development of materials that address these challenges is urgently required for further advancing the various imaging technologies.

Kawano et al.^[^ ^141^ ^]^ prepared a scintillating hybrid 2D perovskite compounds ((C_6_H_5_C_2_H_4_NH_3_)_2_PbX_4_). The self‐organized multiple quantum wells composed of the inorganic component and separated by organic spacers enabled the demonstration of high LY (14 000 photons MeV^−1^, with excellent linearity to *γ*‐rays with energy in the range 122–662 keV) and short decay time (≈11 ns) (see Table 8). This level of performance is slightly higher than that of a common inorganic material such as GSO:Ce. The improved characteristics were ascribed to the 2D nature of the perovskite. On the other hand, the weak energy resolution (FWHM/*E*, where *E* is the energy corresponds to the specific *γ*‐ray induced transition) was attributed to the nonuniform nature of the active layer. Once again, the results highlighted the potential of MHPs as promising scintillating materials for *γ*‐ray detection. Some of the key benefits of perovskite‐based *γ*‐ray detectors over commercial technologies are listed in **Table** **9**.

**Table 9:** Important attributes of perovskite‐based *γ*‐ray detectors over commercially available material technologies

High temperature operation (i.e., no need to operate at cryogenic temperatures). Higher bulk resistivity and thus improved *γ*‐ray detection characteristics. Lower cost and compatibility with temperature sensitive readout electronics. Higher *μ*_h/e_ × *τ* product than TlBr‐based detectors. Higher *γ*‐ray stopping power than commercially available CdTe detectors.

## 4 Summary and Future Perspective

Recent years have witnessed the emergence of MHPs as a promising family of materials for application in next‐generation X‐ray and *γ*‐ray detector technologies. Noticeably, the field has skyrocketed since 2017, as evidenced by the volume of publications and relevant citations received to date (Figure 3). This global interest stems from the intriguing physical properties that these synthetic perovskites possess, which, when combined with advanced device engineering, can yield detectors with performance characteristics on par, or in some cases even superior, to those of incumbent technologies.

The two types of high‐energy radiation detectors that MHPs have been utilized to date are the direct and indirect detector technologies. Direct detectors are simpler to manufacture than scintillation systems, which are bulkier and less portable. Depending on the application, however, the use of scintillation detectors can address specific challenges that direct X‐ray detectors face. This is why significant ongoing research focuses on improving scintillation systems further in terms of their manufacturability, spatial resolution, response time, and energy resolution. To this end, MHPs have demonstrated the potential to address many of the shortcomings, including manufacturability, sensitivity, and response time to the degree that numerous materials are quickly becoming competitive to commercial technologies such as NaI and CsI.

MHPs have also demonstrated tremendous potential for direct X‐ray detection. The outstanding performance of numerous perovskite‐based devices reported to date is mainly attributed to the superb *μ*_h/e_ × *τ* product (1.1 × 10^−2^ cm^2^ V^−1^), long carrier diffusion lengths (up to 175 µm), faster response time, higher sensitivity, high conversion efficiencies, emission in the visible region of the electromagnetic spectrum, quantum confinement (low‐dimensional perovskites), and the characteristically low trap density of states (10^8^ cm^−3^) even in polycrystalline systems processed via inexpensive solution‐phase deposition techniques.

Despite the advantages demonstrated by the perovskite‐based high‐energy radiation detectors, however, there are still many challenges to be addressed before the technology can be commercialized. First, in the case of direct X‐ray perovskite detectors, the resistivity of the active layers (films, crystals, etc.) needs to be increased further in order to reduce the dark current. The use of thicker active layers enables the application of larger bias voltages, which improve the charge collection efficiency but often with adverse effects on electrical noise (dark current) and operational stability due to the field‐induced ion‐migration—this is especially true for polycrystalline films. Possible approaches that can be exploited to overcome these bottlenecks include the use of higher quality single crystals and/or application of larger bandgap perovskites. Use of metallic or doped graphene layers to suppress ion‐migration, have also been reported, as was the utilization of alternative inorganic single‐crystal perovskites.

The second challenge is to enhance the perovskite‐based X‐ray detector's chemical stability toward the ambient atmosphere. The use of all‐inorganic perovskites was shown to address this challenge, although simple device encapsulation could also provide a more tangible solution for commercial applications. The simultaneous enhancement of LY, through improved quantum confinement, and environmental/operational stability, on the other hand, could be achieved by developing advanced low dimension perovskites (e.g., 0D, 1D, 2D). Recent reports show that incorporation of such low dimension perovskite crystals reduces the ion‐migration, hence enabling the application of higher bias across the crystals, while improving the operational stability of the devices.

Increasing the *μ*_h/e_ × *τ* product through crystal engineering of the perovskite layer/crystal is another challenging aspect. The use of indirect bandgap perovskites that exhibit longer carrier lifetimes could be exploited as a possible solution. In sandwich‐type direct X‐ray detectors, introducing conducting materials, such as graphene or other 2D materials, in the charge transporting layer could enhance the conductivity and improve the charge collection efficiency. A further challenge that would also need to be addressed in the near future is the toxicity of Pb present in most MHP compounds studied to date. Substitution of Pb with other high *Z* elements, which are essential for obtaining high X‐ray stopping power, has already yielded encouraging results with plenty of room for further improvements.

The development of colloidal inorganic perovskites as candidates for high‐performance scintillators also appears attractive for deployment in inexpensive, printable, and highly efficient X‐ray and *γ*‐ray detectors. To this end, the vast majority of perovskite materials studied to date exist in their solid form, while reports on colloidal perovskite scintillators are rare. There is no doubt, however, that for various emerging applications of the future, solution‐processable scintillators could enable the realization of affordable X‐ray imaging systems that offer high performance and attractive form‐factor that current technologies lack. For this to materialize, however, more work is needed on materials science and device engineering.

Perovskite‐based *γ*‐ray detectors share the same challenges as X‐ray detectors. In addition to these, the energy resolution of MHPs‐based *γ*‐ray detectors needs to advance further. Improving the uniformity and overall quality of the active layer(s), or single crystal, would certainly help to resolve some of the outstanding issues as would the development of improved materials. Despite the remaining hurdles, however, the future of the perovskite‐based X‐ray and *γ*‐ray detectors appears bright. Only time will tell whether MHP‐based high‐energy radiation detectors would eventually make it to commercial applications ranging from homeland security and medicine to portable radiological identification and energy harvesting devices for space applications.

## Conflict of Interest

The authors declare no conflict of interest.
